# The Involvement of Smooth Muscle, Striated Muscle, and the Myocardium in Scleroderma: A Review

**DOI:** 10.3390/ijms231912011

**Published:** 2022-10-09

**Authors:** Ioana Bratoiu, Alexandra Maria Burlui, Anca Cardoneanu, Luana Andreea Macovei, Patricia Richter, Gabriela Rusu-Zota, Ciprian Rezus, Minerva Codruta Badescu, Andreea Szalontay, Elena Rezus

**Affiliations:** 1 Department of Rheumatology and Physiotherapy, “Grigore T. Popa” University of Medicine and Pharmacy, 16 University Street, 700115 Iasi, Romania; 2 Department of Pharmacology, “Grigore T. Popa” University of Medicine and Pharmacy, 16 University Street, 700115 Iasi, Romania; 3 Department of Internal Medicine, Faculty of Medicine, “Grigore T. Popa” University of Medicine and Pharmacy, 16 Universitatii Street, 700115 Iasi, Romania; 4 Department of Psychiatry, “Grigore T. Popa” University of Medicine and Pharmacy, 16 University Street, 700115 Iasi, Romania

**Keywords:** systemic sclerosis, myositis, smooth muscle, skeletal muscle, sarcopenia

## Abstract

Systemic sclerosis (SSc) is a complex autoimmune disease characterized by heterogeneous changes involving numerous organs and systems. The currently available data indicate that muscle injury (both smooth and striated muscles) is widespread and leads to significant morbidity, either directly or indirectly. From the consequences of smooth muscle involvement in the tunica media of blood vessels or at the level of the digestive tract, to skeletal myopathy (which may be interpreted strictly in the context of SSc, or as an overlap with idiopathic inflammatory myopathies), muscular injury in scleroderma translates to a number of notable clinical manifestations. Heart involvement in SSc is heterogenous depending on the definition used in the various studies. The majority of SSc patients experience a silent form of cardiac disease. The present review summarizes certain important features of myocardial, as well as smooth and skeletal muscle involvement in SSc. Further research is needed to fully describe and understand the pathogenic pathways and the implications of muscle involvement in scleroderma.

## 1. Introduction

Systemic sclerosis (SSc) is a connective tissue disease defined by a complex pathogenesis that includes vascular damage, immune system impairment, and fibrosis of the skin and internal organs [1,2]. The two main subsets of the disease (the limited cutaneous SSc–lcSSc, and diffuse cutaneous SSc–dcSSc) share numerous features, yet differ in terms of prognosis, with the diffuse form being associated with a higher risk of morbidity and mortality [1,3,4,5]. The most widely studied clinical changes of the disease include Raynaud’s phenomenon, digital ulcers, cardiopulmonary and kidney involvement, and digestive manifestations [6,7,8].

Muscle involvement in scleroderma is complex and affects both smooth muscle (such as vascular and digestive smooth muscle) and skeletal muscle [9]. In recent years, the knowledge of the cellular and molecular mechanisms underlying the heterogeneous involvement of the smooth muscle has considerably improved. The new methods of analyzing smooth muscle cells from the vascular wall or gastrointestinal tract have facilitated a better understanding of the clinical aspects [10,11,12]. Regarding skeletal muscle involvement, it has been shown that in SSc it is represented either by myositis, or by non-inflammatory myopathy, both being important debilitating factors for SSc patients [13,14].

The pathogenesis and implications of smooth and striated muscle involvement in scleroderma remains poorly studied compared with other types of organ involvement [15,16]. The present review summarizes certain important features of smooth muscle, skeletal muscle, and myocardial involvement in SSc.

The search of the published data was performed on the EMBASE, EBSCO and PubMed databases, combining the following terms: “systemic sclerosis” or “scleroderma” with “smooth muscle”, “skeletal muscle”, “gastric wall”, “intestinal wall”, “muscle biopsy”, “myopathy”, “myositis”, “anti-PM-Scl antibodies”, “scleromyositis”, “sarcopenia”, “endomyocardial biopsy”, and “cardiac magnetic resonance”.

## 2. Smooth Muscle Involvement

### 2.1. Vascular Smooth Muscle

SSc is defined by a widespread vasculopathy existing before the onset of tissue fibrosis [5,9,17]. It is one of the main pathogenic features involved in the development of Raynaud’s phenomenon, digital ulcers, pulmonary arterial hypertension, and scleroderma renal crisis [6,18,19]. The failure to repair the local vascular injury leads to a chaotic deposition of extracellular matrix [10]. The progressive tissue fibrosis that follows the microvascular impairment involves the appearance of profibrotic myofibroblasts [20]. Myofibroblasts have contractile properties and originate from the vascular endothelial cells through an endothelial-to-mesenchymal transition (EndoMT), a process that is triggered by molecules such as TGF-β (transforming growth factor β), ET-1 (endothelin-1), IL1-β (interleukin 1-β), TNF-α (tumor necrosis factor-α), and other pro-inflammatory cytokines [12]. These unique cells are characterized by particular functions and immunophenotypic aspects: a reduction in their endothelial-specific indicators and the gain of myofibroblast-specific products such as α-SMA [21,22,23]. Therefore, EndoMT determines an aberrant architecture of the microvascular network, with capillary loss and dermal fibrosis [5,9]. Furthermore, the EndoMT process is not exclusively a skin process, as it has also been detected in the lungs, gastrointestinal wall, myocardium, and kidneys [23,24,25].

The vascular tunica media contains smooth muscle cells (VSMCs) that regulate the blood vessel tone and pressure [26,27]. Fully differentiated VSMCs are characterized by a contractile phenotype and the expression of normal contractile proteins, maintaining a quiescent form [28]. However, as a response to specific stimuli (environmental, inflammatory, growth, or mechanical factors) they may switch to a synthetic phenotype, developing into proliferative/migratory cells and extracellular matrix component (ECM) producers [27]. During this change, the expression of α-SMA and smooth muscle myosin heavy chain is downregulated, while other markers are newly acquired (non-muscle myosin heavy-chain isoform-B, cellular retinol-binding protein-1, platelet-derived growth factor-A, collagen type I, and others) [28]. This transformation was described as responsible for the development of atherosclerosis or systemic hypertension [27,29,30]. In SSc, this mechanism is present as well, contributing to the extensive tissue fibrosis that follows the initial vasculopathy [9].

Altorok et al. described a novel method for the isolation of VSMCs from the punch-skin biopsy. The authors investigated six subjects, three with late dcSSc and three healthy controls, and observed an increased proliferation rate, with higher metabolic activity, resistance to apoptosis, and longer viability of VSMCs from SSc patients compared with the control group. Therefore, the altered VSMCs determine uncontrolled vascular tone, and intimal and media proliferation, finally leading to subsequent fibrosis [31].

It has been stated that Th17 (T helper 17) cells are involved in the pathogenesis of SSc [32]. In addition, a study reported the proliferative role of IL-17A (interleukin 17A) on dermal VSMCs in SSc, causing increased collagen synthesis and secretion [33]. Following this statement, studies were carried out regarding the inhibition of IL-17A as a potential therapeutic target in SSc, but more research is needed to elucidate the extent to which IL-17 blockade could influence SSc manifestations [34,35].

The mesenchymal stromal cells (MSCs)—the main source of VSMCs—possess unique alterations in their differentiation process as a response to specific growth factors (connective tissue growth factor, CTGF; basic fibroblast growth factor, b-FGF; platelet derived growth factor BB, PDGF-BB; and transforming growth factor β1, TGF-β1). An in vitro study showed that the MSCs collected from SSc patients differentiated mainly into myofibroblasts, with uncontrolled contractile VSMCs conversion. Before adding the growth factors, the MSCs from healthy controls and SSc patients had a comparable phenotype with irregular shapes and α-SMA. In response to CTGF, the SSc-MSCs failed to differentiate into normal contractile VSMCs, while b-FGF promoted the synthetic phenotype. Moreover, in SSc, TGF-β1 generated more myofibroblast differentiation compared with healthy controls [36].

Notably, pulmonary arterial hypertension is one of the most severe and potentially life-threatening complications in SSc [37,38]. The VSMCs’ activity also plays an important role in the development of pulmonary arterial hypertension through the same conversion from a contractile phenotype to a synthetic one [28,39]. The presence of reactive oxygen species at the level of pulmonary arterial muscle cells may favor collagen synthesis. After exposure to the sera from patients with SSc-associated pulmonary arterial hypertension, pulmonary arterial smooth muscle cells exhibited an oxidative stress-related enhancement in collagen production [40,41]. Moreover, the level of leukotriene B_4_ was found to increase in patients with SSc and pulmonary arterial hypertension [42]. The presence of leukotriene B_4_ was shown to determine hypertrophy and increased the proliferation of the pulmonary arterioles’ smooth muscle cells [43].

The pulmonary vascular smooth muscle impairment is associated with the dysregulation of transcription factors such as Krűppel-like factor 5 (KLF5). These factors play a key role in the proliferation and apoptosis of VSMCs in pulmonary hypertension [44]. Complex autoimmune and inflammatory mechanisms were investigated as part of the pulmonary arterial hypertension pathogenesis, along with autoantibody production and genetic abnormalities [39,45,46]. However, the relationship between inflammation, the immune system and the pulmonary wall remains a matter of further investigation.

The VSMCs’ dysfunction and the differentiation from fibroblast to myofibroblast involves the PDGFR (platelet-derived growth factor receptor) molecular pathway [28]. A recent study indicated the decisive role of macrophage-derived PDGF-B in the pathological development of the smooth muscle cells [47]. High levels of PDGF, PDGFR-β, and anti-PDGFR-α antibodies were also identified in SSc patients’ skin [48,49]. A study published in 2017 evaluated the in vitro effects of these molecules on human smooth muscle cells of 11 SSc patients. After exposing the smooth muscle cells to anti-PDGFR-α antibodies from SSc patients’ sera, the smooth muscle cells converted into a synthetic phenotype, with all the aforementioned properties [41,50].

When combined with IgG from SSc patients’ sera, VSMCs were shown to exhibit a higher growth index and develop a profibrotic response through the activation of various protein kinases that adjust the vascular remodeling process [51]. Moreover, studies reported the presence of VSMC antibodies in SSc patients’ sera, irrespective of pulmonary hypertension. These antibodies alter VSMCs’ contraction and are directed against STIP1 (stress-induced phosphoprotein 1) and α-enolase [52].

### 2.2. Digestive Smooth Muscle

#### 2.2.1. Esophageal Involvement

Some of the most common clinical features in SSc patients result from esophageal involvement, which leads to dysphagia and gastroesophageal reflux disease (GERD) [53,54]. Esophageal dysfunction may appear in approximately 90% of SSc patients [55,56,57]. In a very early stage, the clinical symptoms may be absent, although esophageal involvement can be present at a histological level [58,59]. Smooth muscle atrophy and fibrosis play an important role in the appearance of esophageal dismotility. This results in reduced pressure and the absence of coordination in the lower esophageal sphincter, together with an inefficient peristaltic pump [6,58]. In the beginning, vasculopathy may generate irreversible muscle and nerve lesions. Similar to Raynaud’s phenomenon, the vasospasm decreases the muscle blood supply, leading to motor impairment, and platelet and endothelin activation as a response to ischemia [60,61]. As a consequence, there is a disproportionate extracellular matrix formation and aberrant collagen deposition [55,61].

A case–control study evaluated the esophageal biopsy samples of 74 patients with SSc post-mortem. The authors found important histological findings in the smooth muscle layer in over 90% of the samples. Atrophic areas observed in the circular smooth muscle layer were present in all subjects. In 66% of the samples, atrophic modifications in the longitudinal layer were also present. Moreover, the atrophy was more severe in the circular layer, although there was no correlation between the disease duration and the degree of atrophy. In some cases with diffuse esophagitis, inflammatory cells were also present in the muscular layer of the esophagus [62].

Another study that included the endoscopic and endosonographic examination of the esophagus in 25 patients with SSc observed a thicker esophageal wall compared with healthy controls. Moreover, the esophageal muscularis layer and submucosa were thicker in SSc patients that suffered from dysphagia. The study did not identify any correlation between disease duration and the structure and width of the esophageal wall [63].

Taroni et al. published a histological and molecular overview of the esophageal changes in scleroderma. The authors identified three molecular subsets: inflammatory, proliferative, and non-inflammatory signatures. They are different from the classical clinical subtypes and do not depend on disease duration, antibodies, skin score, or collagen deposition. The study suggested the possibility of an inflammatory mechanism as part of the esophageal involvement, since interferon-induced proteins and inflammasomes were detected in the inflammatory subset [64].

#### 2.2.2. Gastric Involvement

Gastric involvement appears in approximately 50% of patients with scleroderma [65] and is characterized by the presence of GAVE (gastric antral vascular ectasia), gastric hypomotility, and gastroparesis. The most common symptoms of gastric involvement include early satiety, bloating, regurgitation, nausea, vomiting, eructation, and abdominal pain [66]. Gastric dysmotility leads to prolonged gastric emptying and is reflected by altered myoelectric activity [67]. The histological modifications of the gastric wall were previously described in various studies (Table 1).

#### 2.2.3. Lower Digestive Tract Involvement

Small bowel involvement includes dysmotility, leading to chronic pseudo-obstruction or small intestinal bacterial overgrowth (SIBO). Patients often present with abdominal distention, nausea, diarrhea, bloating, abdominal pain, and other symptoms due to malabsorption [67,70,71]. Colonic involvement can be suggested by the presence of alternating constipation with diarrhea, fecal incontinence, steatorrhea, or such potentially life-threatening complications as colonic volvulus or perforation [67,72].

Several studies showed there is a progressive fibrosis of the bowel smooth muscle and vasculopathy that promotes bacterial overgrowth [73]. Moreover, there is a defective contractile function, with reduced power and decreased velocity of the smooth muscle cells that combines with the stiffness of the tissue [74]. There is also an increased collagen deposition in the mucosa, submucosa and muscularis, especially in the large intestine. The reduced colonic contractility as a response to a cholinergic agonist was demonstrated using a scleroderma mouse model [75].

A recent study investigated the histological aspect of the intestines in SSc on autopsy segments of the small bowel and colon, which were compared with material from healthy controls. Vascular impairment was more often present in SSc patients, especially in the small bowel. The enteric nervous system was altered, especially in the colon, where neuronal density was decreased in the myenteric plexus, but not in the muscularis propria. The presence of α-SMA (α-smooth muscle actin) protein was no different between SSc and healthy controls. However, more inclusion bodies, an both focal and generalized fibrosis were more often present in the circular layers of SSc patients. Moreover, focal fibrosis was found with a greater frequency in the colon, as well as in the longitudinal layers [76].

Internal anal sphincter involvement may also be present due to neuropathy or myopathy, although it is usually not reported by patients. Studies show that the internal sphincter’s function is altered, with severe hypotonia due to tissue fibrosis and excessive collagen deposition [70,77,78].

## 3. Skeletal Muscle Involvement

Skeletal involvement in SSc can range from unspecific muscle involvement without muscular inflammation to real inflammatory myositis. However, there is a “grey” area with patients who are hard to classify as having one or the other.

Patients may experience non-progressive non-inflammatory myopathy as a consequence of digestive disturbance, malnutrition, a sedentary lifestyle, or contractures of fibrotic skin. In other cases, it appears as an overlapping syndrome with inflammatory myopathy when patients meet the classification criteria for both diseases [16,79,80]. SSc-associated myopathy was also described when patients meeting the SSc classification criteria developed muscle weakness, myalgia, elevated CK (creatin-kinase), abnormal EMG (electromyography), MRI (magnetic resonance imaging), or muscle biopsy [16]. Another term cited in the literature is scleromyositis, referring to patients with anti-PM/Scl autoantibodies and Raynaud’s phenomenon, scleroderma skin changes, arthralgias/arthritis, myalgia/myositis, interstitial lung disease, and/or calcinosis, with increased mortality due to cardiopulmonary involvement [81]. Patients often have marked peripheral muscle weakness in the upper and lower limbs, with reduced range of motion in the shoulders, reduced strength in the lower limbs, and physical disability [82,83].

Moreover, SSc may be associated with idiopathic inflammatory myopathy (IIM). Maundrell et al. reported that myositis antibodies and myositis-specific histological features were more often present in the SSc-IIM group compared with IIM alone, suggesting a possible connection between the two diseases [84].

### 3.1. Systemic Sclerosis and Inflammatory Myopathy

The prevalence of skeletal myopathy varies between 5 and 96%, with differences between the geographic areas [85]. This variation depends also on the criteria that each study used in defining muscle involvement. A recent study showed that SSc patients with a history of exposure to environmental factors, especially silica, had a higher frequency of myopathy [86]. In some cases, the SSc/polymyositis overlap syndrome may not be fully differentiated from SSc-associated myopathy [13,16,87]. Currently, whether the presence of inflammatory skeletal muscle changes should be regarded primarily as a disease-related manifestation or rather as a sign of overlap with IIM remains a matter of debate.

The recognition of muscle involvement in SSc can be based on symptoms, clinical signs, and abnormal elevated values of accessible laboratory tests such as serum CK or aldolase, and should represent a priority in the clinical follow-up, as it is associated with higher mortality and poor quality of life [88,89,90]. The clinical presentation shares symptoms with polymyositis or dermatomyositis, the most frequently reported being myalgia, muscle weakness, and tenderness [91]. The skeletal muscle involvement is usually symmetric in the proximal limbs. Distal weakness can also be present, although it is difficult to differentiate from the consequences of severe skin sclerosis [13]. Although rare, other muscles may also be involved, such as the cervical muscles, as a manifestation of SSc–IIM overlap syndrome [92,93]. Moreover, skeletal and cardiac involvement are associated, patients with myopathy being at risk for congestive heart failure [79].

Regarding the laboratory findings in myopathy, CK and aldolase levels have been studied, the latter having the highest predictive value [80,94]. However, these are sometimes unreliable markers; thus, other examinations are required such as EMG, MRI, or muscle biopsy [88]. EMG is usually altered, with modifications similar to those present in idiopathic inflammatory myopathies [16]. Currently, whole-body MRI is a more common method of investigation as it is non-invasive and can detect the presence of myositis, despite the absence of standard scoring systems. In a cohort of 34 SSc patients, muscle edema was present in 13 of the subjects, of which only 2 had myopathy symptoms, suggesting the importance of imaging to fully evaluate these patients, regardless of the presence of symptoms. Moreover, it was associated with male gender and the diffuse cutaneous phenotype, although no relationship was found between skeletal and cardiac muscle involvement [95]. Furthermore, MRI can be used to evaluate the presence of diffuse fibrosis in the peripheral muscles using extracellular volume determination [96]. Certain key clinical and laboratory findings from studies of skeletal muscle involvement in SSc patients are described in Table 2.

In SSc-associated skeletal myopathy, the histological changes described to date were similar to (or suggestive of) polymyositis, dermatomyositis, immune-mediated necrotizing myopathy, fibrosing myopathy, or non-specific myositis (Table 3) [87]. Lefebvre et al. performed a scoping review including all major studies published between 1955 and 2019 that provided information on the histological features in patients with SSc and myositis. They reviewed 77 studies and summarized 559 muscle biopsies. Inflammatory infiltrates, composed mostly of T cells, were the most prevalent finding in 57% of the biopsies, 49% of them being endomysial, 42% perimysial, and 41% perivascular. In 48% of the biopsies, myofiber atrophy was described, mostly perifascicular. Necrosis of the muscle fibers was reported in 56% of the biopsies and in 41% regenerative fibers were present. The presented data suggest that the predominant features are not specific for SSc-myositis patients, although up to a third of patients present acute neurogenic atrophy and fibrosis [101]. Furthermore, fibrosis was associated with younger age of SSc onset, diffuse cutaneous form, African American population, lower forced vital capacity, positivity for anti-Scl-70 and U3-RNP antibodies, and increased mortality compared with patients with inflammatory myopathy [102].

A recent study reported a specific histological pattern called “minimal myositis with capillary pathology” (MMCP) present in a high percentage of patients with lcSSc. Usually, patients with MMCP have rare organ involvement, mild CK elevation, and mild disease activity, yet no association with SSc-specific antibodies [103].

**Table 3:** Muscle biopsy patterns reported in patients with SSc and muscle involvement symptoms.

Author	Patients	Findings
Paik et al. [104]	The study included a database of 2830 patients, from which 42 had muscle weakness and underwent a muscle biopsy.	Necrosis and inflammation were the most prevalent findings, accounting for 67% and 48%, respectively. Fibrosis was present in 33% of the biopsies. A marker for acute neurogenic atrophy (esterase positivity of angular atrophic fibers) was described in 48% of the muscle biopsies. The most common patterns found were non-specific myositis in 38.5% of the cases and necrotizing myopathy in 21.4% of the biopsies.
Corallo et al. [15]	The study included 35 patients with clinical, biological, and EMG exams of muscle involvement.	The most common patterns found included fibrosis in 81% of cases and microangiopathy in 92% of the biopsies. In 85% of cases there was no inflammatory infiltrate. They also described loss in small endomysial vessels and complement deposition in endomysial capillaries.
Siegert et al. [103]	From the 367-patient cohort, 18 patients underwent a muscle biopsy due to the presence of muscle involvement symptoms.	In 12 biopsies, a unique pattern was described as “minimal myositis with capillary pathology”, characterized as a mild myositis with rare histopathological alterations. The rest of the biopsies were very different: one had typical anti-synthetase syndrome alterations and five were non-specific.

From the serology markers, it can be seen that anti-PM/Scl antibodies were associated with a distinct phenotype, muscle involvement being reported more frequently in PM/Scl-positive patients [80,105]. In SSc, the prevalence of anti-PM/Scl antibodies varies between 3 and 17.5% as highlighted in Table 4 [105,106]. They are directed against two main autoantigenic proteins, PM/Scl-75 and PM/Scl-100. These antibodies are not specific for SSc as they can be found in polymyositis, dermatomyositis, and SSc-polymyositis overlap syndrome [107,108]. Patients with anti-PM/Scl antibodies developed muscle weakness and interstitial lung disease as the disease progressed. Compared with patients with the antisynthetase syndrome, dermatomyositis, and immune-mediated necrotizing myopathy, they have been more often diagnosed with the following in the order of their prevalence: “mechanic’s hands”, Raynaud’s phenomenon, sclerodactyly, telangiectasias, esophageal reflux disease, subcutaneous edema, puffy hands, and calcinosis [109]. Moreover, positivity for these antibodies was correlated with pulmonary fibrotic patterns and cardiac complications [110].

*Scleromyositis* is a term proposed for the SSc-IIM overlap syndrome, characterized by the presence of both classification criteria of SSc and myositis, using the 2013 ACR/EULAR classification criteria for SSc and the 2017 EULAR/ACR classification criteria for idiopathic inflammatory myopathies [116,117,118]. It is also the most frequent, accounting for almost 44% of all other SSc overlap syndromes [116]. It is also the most frequent SSc overlap, accounting for almost 44% of all SSc overlap syndromes [116].

A recent study that included 42 patients with scleromyositis hypothesized the possibility of distinct subsets of scleromyositis based on the autoantibody profile. The subjects were divided into three groups based on the presence of SSc-specific, SSc-overlap, or no SSc-related antibodies, where SSc-specific antibodies were considered anti-centromere, anti-topoisomerase-I, anti-RNA-polymerase-III, anti-Th/To, and anti-fibrillarin, and the antibodies indicative of SSc-overlap were anti-PM/Scl, anti-Ku, and anti-U1RNP antibodies. The authors found that skin thickening was absent in 50% of the SSc-overlap group and in 30% of the no SSc-related group. Proximal muscle weakness was present in more than 70% of all of the groups, while distal weakness was present in 57% of the SSc-overlap group and in 55% of the no SSc-related group. Moreover, head drop/bent spine, elevated CK levels and other overlap features such as Raynaud’s phenomenon, calcinosis, and arthritis were more frequent in scleromyositis.

Regarding histopathology features, perifascicular atrophy, necrosis, inflammation, and neurogenic changes were described in the SSc-overlap group [119]. Furthermore, in a Hungarian cohort, the genetic features HLA-DRB1 and DQA1 and dysphagia were more common in overlap patients, while some characteristics present at the onset of SSc, such as fever, subcutaneous calcinosis, heart involvement, and claw hand deformity, were correlated with pulmonary arterial hypertension in this group [120,121]. A group of authors also described a seronegative form of scleromyositis, with the absence of SSc-specific or SSc-overlap antibodies, and explored the non-ACR/EULAR features that led to a positive diagnosis of scleromyositis. The authors hypothesized that myositis might represent an early SSc manifestation since it was present in 55% of the subjects as the first non-Raynaud manifestation. Moreover, skin involvement was absent in almost half of the patients at the time of myositis diagnosis, while several features (Raynaud’s phenomenon, ANA, SSc pattern in capillaroscopy, and lower esophageal dysmotility) were established as possible indicators for a scleromyositis diagnosis [122].

### 3.2. Sarcopenia

SSc patients have impaired muscle function associated with fatigue and disability that can be sometimes a consequence of a natural phenomenon called sarcopenia. Although it is mainly found in the older population, sarcopenia may also be described in certain chronic diseases, irrespective of age [123,124]. Several studies have proved that chronic inflammation is an important risk factor for sarcopenia. Systemic inflammation may significantly contribute to the appearance of sarcopenia in chronic immune-inflammatory diseases such as scleroderma [125,126]. Furthermore, SSc disturbs the patients’ physical status and nutrition, being a major risk factor for sarcopenia, especially in cases of longer disease duration [123,127]. The EWGSOP (European Working Group on Sarcopenia in Older People) proposed a definition of sarcopenia in 2010, as well as a revised definition and diagnosis criteria in 2019. In the latter version, low muscle strength indicates probable sarcopenia, low muscle quantity or quality confirms the diagnosis, and, if low physical performance is also present, sarcopenia is regarded as severe [128].

The prevalence of sarcopenia in SSc varies between 15% and 53%, depending on the definition and the analyzed group of subjects. A number of studies use only the SMI (skeletal muscle index—a tool for the assessment of muscle mass), while others included an evaluation of muscle strength (the handgrip strength index), as described in Table 5 [129,130,131,132,133,134].

## 4. Cardiac Muscle Involvement

Recent studies suggest that the risk of cardiovascular disease in SSc is higher compared with the general population [135]. Moreover, according to the EUSTAR database analysis, it accounts for 27% of the deaths [136]. Apart from the traditional cardiovascular risk factors, there are a series of other risk factors that are associated with the primary myocardial disease: male gender, advanced age, the diffuse form of SSc, muscle involvement, digital ulcers, rapid skin progression, and the presence of anti-topoisomerase I antibodies [137,138,139]. Cardiac involvement in SSc is heterogenous depending on the definition used in the various studies, ranging between 80% when defined from the histopathological point of view, and 5% when defined from the clinical point of view [140,141]. The majority of SSc patients experience a silent form of cardiac disease [142,143].

Besides the myocardium, the pericardium, conduction system, and cardiac valves may also be involved [139]. The cardiac manifestations include a wide range of clinical possibilities, including myocardial fibrosis, ventricular systolic and diastolic dysfunction, myocarditis, congestive heart failure, pericardial disease, arrhythmias, conduction defects, and microvascular dysfunction [139,140,144].

The pathogenesis of cardiovascular involvement is very complex, beginning with microvascular and immune dysfunction. The cardiac Raynaud’s phenomenon and the collagen deposition that lead to hyperplasia of the intramural arteries cause intermittent ischemia. As a consequence, areas of necrosis develop that will be replaced by myocardial fibrosis [139,144,145]. Patchy myocardial fibrosis is considered the hallmark of myocardial involvement in SSc. It is present in both ventricles and does not correlate with the distribution of coronary arteries [139,146]. Histopathological patterns of the myocardium involvement in SSc are described in detail in Table 6, from the first reported autopsies to the most recent endomyocardial biopsies.

For a proper diagnosis of myocardial involvement in SSc, various investigations are being used to assess cardiac injury, such as cardiac biomarkers, electrocardiography, transthoracic echocardiography, cardiac magnetic resonance (CMR), nuclear techniques, or endomyocardial biopsy [139,144,146]. CMR is one of the most accurate non-invasive and non-irradiating methods used to evaluate myocardial inflammation and fibrosis, being able to identify cardiac disease through specific indexes and measurements. High T2 signal values are used to diagnose myocarditis, indicating myocardial edema. LGE (late gadolinium enhancement) measures focal fibrosis and is frequently found in SSc patients in a nonischemic pattern. Moreover, T1 mapping and ECV (mean extracellular volume) quantification are a way of measuring the relaxation and expansion of the myocardial tissue with values that are significantly higher in SSc [144]. Studies that evaluated SSc patients using CMR are described in detail in Table 7.

## 5. Conclusions

The muscle involvement in SSc includes, to a variable extent, both smooth and skeletal muscle. The altered VSMCs acquire higher metabolic and proliferative rates, leading to important remodeling of the vessel wall. Very few studies have managed to isolate VSMCs from SSc patients and describe the intimal and media proliferation together with the final fibrosis stage. This conversion is clinically translated into Raynaud’s phenomenon, digital ulcers, pulmonary arterial hypertension, and scleroderma renal crisis.

GI dysmotility develops through a staged process where different pathophysiological mechanisms overlap, leading to the clinical forms that the clinician sees. Progressive vasculopathy and myopathy, a neurological disorder of the neuromuscular junction, or the presence of antimyenteric antibodies appear to be the most common theories for the mechanism of GI dysfunction.

Skeletal involvement is often part of the onset symptoms of the disease, causing significant disability and low quality of life together with a poor prognosis. Regardless of the diagnosis method used (electromyography, MRI, elevated CK levels, or muscle biopsy), fatigue, muscle weakness, and myalgia are the most common symptoms. Furthermore, the impaired muscle function can lead to secondary sarcopenia.

Heart involvement in SSc is heterogenous depending on the definition used in the various studies. The majority of SSc patients experience a silent form of cardiac disease.

Systemic sclerosis is a complex autoimmune disease characterized by heterogeneous changes involving numerous organs and systems. The currently available data suggest that muscle injury (both smooth and striated muscles) is widespread and leads to a number of notable clinical manifestations. Nevertheless, further research is needed to fully describe and understand the pathogenic pathways and the implications of muscle involvement in scleroderma.

## Figures and Tables

**Table 1:** Histological aspects of the gastric wall in systemic sclerosis.

Authors	Study Participants	Results
Ibba-Maneschi et al. [68]	One patient with lcSSc with severe digestive involvement. The sample was obtained after total gastectomy.	- Important fibrosis among the smooth muscle cells with disorganized myofilaments and thickened dense bodies. - Nerve endings covered in elastic and collagen fibers, disconnecting them from the smooth muscle cells. - Erythrocytes and neutrophils partially or completely clogged the vascular lumen.
Manetti et al. [24]	Eight patients with dcSSc and six with lcSSc all with digestive involvement. The samples were obtained through endoscopic gastric biopsy.	- Extracellular matrix components were progressively present in the lamina propria and muscularis mucosae. - The smooth muscle cells were encircled by focal fibrosis and areas of atrophy and normal muscle tissue were present in the same section. - Type I and type III collagen was stocked in the lamina propria, muscularis mucosae, and muscle layer. - Myofibroblast presence was identified in the muscularis mucosae and muscle layer similar to the smooth muscle atrophy areas. - Profibrotic factors (TGFβ, CTGF) were present in all the layers of the gastric wall.
Manetti et al. [69]	Two patients with dcSSc and one with lcSSc, all with severe digestive involvement who underwent gastric surgery.	- Collagen deposition was present in the lamina propria and muscularis mucosae. - Focal fibrosis was seen around the smooth muscle cells with alternating areas of atrophy and normal muscle cells with disorganized myofilaments and thickened dense bodies. - The myenteric plexus was covered in collagen bundles.
Zuber-Jerger et al. [63]	The study included 25 patients with SSc (14 with dcSSc and 11 with lcSSc) and 25 controls. The samples were obtained through gastric endoscopy.	- The thickness of the gastral corpus layers did not differ between the two groups. - The antral muscularis and submucosa were thicker compared with controls. - The thickness of the gastric wall was correlated with the presence of dysphagia, but not with the subtype of SSc or disease duration. - The duodenal wall, submucosa, and muscularis were thicker in patients with a greater Medsger disease severity score, but with no association with disease duration or subtype.

TGFβ—transforming growth factor β; CTGF—connective tissue growth factor.

**Table 2:** Skeletal muscle involvement in SSc as reported by previous studies: clinical and laboratory findings.

Author	Patients	Myopathy Diagnosis	Muscle Involvement	Other Findings
Ranque et al. [91]	A total of 40 patients with SSc, of which 30 with dcSSc and 80 matched healthy controls.	The presence of muscle weakness or myalgia, more than five times the normal value of CK associated with muscle involvement in at least one of the following investigations: EMG, MRI, or muscle biopsy.	A total of 78% had muscle weakness, 83% had myalgia, and CK was increased in 82% of the patients. Among the 17 subjects who underwent an EMG, 94% had a myopathic pattern. Of the 13 patients who received an MRI, 77% showed specific alteration. A total of 35 patients underwent a muscle biopsy and the results were published previously [97].	After multivariate analysis, the reduced FVC < 75%, heart involvement, and the absence of anti-centromere antibodies were associated with myopathy.
Tolédano et al. [94]	A total of 137 patients with SSc were included, 31% having a diffuse form.	The presence of muscle weakness was defined as a decrease of 10% of the maximal score from baseline at the MMT-8 score and muscle involvement in at least two of the following exams: EMG, MRI, or muscle biopsy.	A total of 56% of the patients presented myalgia. Fourteen patients described proximal muscle weakness and nine had diagnosed myopathy according to the mentioned criteria.	Patients with aldolase levels greater than 9 U/L were at risk of developing associated myopathy.
Schanz et al. [98]	A total of 18 patients were enrolled, 15 with dcSSc.	The presence of myalgia or muscle weakness. All of the patients were given an MRI.	Muscle weakness was present in 28% of the patients, while myalgia was found in 17% of them. A total of 47% of the subjects had elevated levels of CK. Muscle edema and hyperemia were present in 78% of the patients.	Muscle weakness was positively correlated with edema, hyperemia, and perifascial enhancement on MRI.
Jung et al. [89]	A total of 1043 patients were included, of which 396 had dcSSc.	The presence of elevated levels of CK (≥200 μ/L for women and ≥250 μ/L for men) and a positive myositis/myopathy history documented by a physician.	Of the subjects included, 5.6% had elevated CK levels and 9.7% had a history of myositis/myopathy	The patients with elevated CK were more frequently males, younger, with tendon friction rubs, with an FVC < 70%, and a higher HAQ-DI score. Moreover, the ribonucleoprotein and Scl-70 antibodies were more frequently positive in these patients.
Paik et al. [90]	A total of 1718 subjects were included.	The muscle involvement was defined by the Medsger muscle severity score. Muscle weakness means a score greater than 1.	There were 392 patients diagnosed with muscle weakness. Of them, 24% had a diagnosis of polymyositis/dermatomyositis from which 47.8% also had a muscle biopsy.	The patients from the weak group had more frequent dcSSc, a higher mRSS score, a shorter disease duration from the first non-Raynaud phenomenon, and more frequent renal crisis. Moreover, they had a higher CK level, more severe digestive and heart involvement.
Zhou et al. [99]	A total of 204 patients were included.	Symptoms of muscle involvement (fatigue, muscle weakness, and myalgia) and at least one of the following: elevated CK levels (≥145 U/L), pathologic muscle biopsy, EMG, or MRI revealing inflammatory changes.	A total of 44 patients were diagnosed with myopathy, all having elevated CK levels. Muscle involvement symptoms were used for diagnosis in 24 cases, while 20 underwent an EMG, MRI, or muscle biopsy as follows: 12–EMG; 4–MRI; and 4–muscle biopsy.	Patients diagnosed with myopathy more often had dcSSc, interstitial lung disease, digestive involvement, digital ulcers, a higher mRSS score, and a higher Valetini disease activity score compared with those without myopathy. In the myopathy group the incidence of pulmonary hypertension and pericardial effusion was also higher.
Baumberger et al. [100]	A total of 454 patients were investigated, from which 58 fulfilled the myopathy criteria and were further compared with the other 58 SSc patients without myopathy.	At least one of the following: elevated levels of CK or aldolase, proximal muscle weakness documented by a physician, muscle atrophy on physical exam, or positive myositis autoantibodies.		In the myopathy group, scleredema, tendon friction rubs, dysphagia, and cardiac arrhythmias were found more frequently, with patients having a greater disease activity score. Moreover, the anti-PM-Scl antibodies and elevated levels of CK and Troponin T were found more often in the myopathy group compared with non-myopathy-SSc patients.

CK—creatine kinase; EMG—electromyography; MRI—magnetic resonance imaging; FVC—forced vital capacity; MMT—manual muscle testing grading system; mRSS—modified Rodnan skin score; HAQ-DI—Health Assessment Questionaire Disability Index.

**Table 4:** Studies that tested the presence of anti-PM/Scl antibodies in SSc patients.

Author	Patients	Findings
D’Aoust et al. [111]	The study included 763 patients with SSc; 62% with lcSSc, and 38% with dcSSc.	Anti PM-1-α antibodies were present in 55 patients, of which almost 50% had no other SSc-specific antibodies. These patients were younger at the onset of the disease, being diagnosed more often with inflammatory myositis, calcinosis, inflammatory arthritis, and overlap disease. However, interstitial lung disease and digestive involvement were present less frequently.
Wodkowski et al. [112]	The study included 1574 patients.	Anti-PM75 antibodies were monospecific in 1% of the patients, and anti-PM100 were monospecific in 0.7%. In 1.7% both antibodies were present. A total of 75% of the anti-PM75 group and 90% of the anti-PM100 group had lcSSc. Inflammatory myositis was present in 36% of the patients with both antibodies, but only in one patient with anti-PM75 antibodies and in none of the patients with anti-PM100 antibodies. High rates of calcinosis were present in the monospecific groups of anti-PM75 and anti-PM100 antibodies.
Wielosz et al. [106]	The study included 126 patients with SSc. After analyzing the presence of anti-PM/Scl antibodies the two groups were clinically compared.	Anti-PM/Scl antibodies were present in 22 patients (17.5%), of which 13 had anti-PM/Scl-100 antibodies and 14 anti-PM/Scl-75 antibodies. In the positive group the incidence of myalgia, myositis and joint contractures was higher. Of the 22 positive patients, 9 had overlap syndrome—7 with polymyositis and 2 with dermatomyositis.
Leurs et al. [113]	The study included 300 patients with SSc, of which 73% had lcSSc.	A total of 17% of the patients had at least one myositis-specific or associated antibody, 8% had at least one myositis-specific antibody, and 9.7% had at least one myositis-associated antibody. Patients with anti-PM/Scl-75 antibodies (3%) more often had ILD and myositis, while those with anti-PM/Scl-100 antibodies more often had digital ulcers and myositis.
Lazzaroni et al. [114]	The study included 7353 patients from the EUSTAR database.	Anti-PM/Scl antibodies were present in 295 (4%) patients, of which 144 had only anti-PM/Scl antibodies. Their presence was associated with male sex, proximal muscle weakness and atrophy, CK elevated levels, and lung fibrosis. Of the 144 patients with anti-PM/Scl antibodies, 5.56% had a scleroderma renal crisis, as they were more exposed to glucocorticoids. Furthermore, elevated CK levels were correlated with cardiac involvement, ILD, intestinal dysfunction, joint contractures, and tendon friction rubs.
Iniesta Arandia et al. [115]	The study included 1797 patients with SSc from RESCLE, from which 947 patients were tested for anti-PM/Scl antibodies.	Of the included patients, 7.6% were positive for anti-PM/Scl antibodies. Puffy fingers and arthralgias were more common than Raynaud’s phenomenon at the onset of the disease. In this group, myositis, arthritis, and interstitial pulmonary disease with more severe ILD were more frequent.

ILD—interstitial lung disease; CK—creatine kinase.

**Table 5:** The presence and clinical significance of sarcopenia in SSc patients.

Author	Year	Participants	Definition of Sarcopenia Used	Results
Doerfler et al. [134]	2017	The study included 18 patients, predominantly women (89%), aged 51 ± 11 years.	RSMI tool was used with the cutoff values of 7.26 kg/m^2^ for men and 5.50 kg/m^2^ for women.	Sarcopenia was present in 53% of the subjects at the beginning of the study and in 39% of the subjects at follow-up, after 6 months of receiving medical nutrition therapy.
Caimmi et al. [129]	2018	The study included 141 patients with a mean age of 63 ± 13 years. Almost 70% had lcSSc.	RSMI tool was used with the cutoff values of 7.26 kg/m^2^ for men and 5.50 kg/m^2^ for women.	Sarcopenia was described in 20.7% of the patients, being more frequent in malnourished patients. Sarcopenia was correlated with lower BMI, longer disease duration, lower predicted DLCO values, higher Medsger severity score for the lungs and skin, and lower physical activity. Disease duration and Medsger severity score were independent risk factors for sarcopenia in a multivariate analysis.
Siegert et al. [130]	2018	The study included 129 patients aged between 28 and 83 years. More than 90% were women and almost 55% had lcSSc.	EWGSOP definition was used with the cutoff values of 7.26 kg/m^2^ for men and 5.50 kg/m^2^ for women.	Sarcopenia was present in 22.5% of the subjects, with no statistical differences for age, disease duration, digestive involvement, and presence of myositis or ILD between sarcopenic and non-sarcopenic patients. Sarcopenia was correlated with lower weight, malnutrition, decreased handgrip strength and knee extension, and lower quality of life.
Corallo et al. [131]	2019	The study included 62 patients aged between 32 and 78 years, of which 81% had lcSSc.	Sarcopenia was defined using the EWGSOP definition. They used the RSMI tool with the cutoff values of 7.26 kg/m^2^ for men and 5.50 kg/m^2^ for women. HGS was measured with a dynamometer.	When using the RSMI tool the prevalence of sarcopenia was 42%. In this case, the condition was associated with disease duration, higher mRSS, esophageal involvement, higher ESR and ANA levels, low DLCO, and a more severe capillaroscopic pattern as the RSMI worsened. When the HGS index was used, the prevalence of sarcopenia was 54.8% and correlated with disease duration, higher mRSS, esophageal involvement, higher ESR and ENA, low DLCO, and capillaroscopic pattern.
Paolino et al. [132]	2020	The study included 43 patients with SSc and 43 age- and sex-matched healthy controls.	Sarcopenia was defined by the EWGSOP definition and the RSMI index was used with the cutoff values of 7.26 kg/m^2^ for men and 5.50 kg/m^2^ for women.	Sarcopenia was diagnosed in 23.26% of the patients, significantly higher compared with healthy controls. The “late” pattern on NVC was correlated with the presence of sarcopenia, compared with “active” or “early” pattern. Sarcopenic patients presented an important loss of capillaries and altered capillary array, lower total/fat/lean mass, higher incidence of digital ulcers, decreased DLCO/VA values, and lower BMD in the trunk and upper and lower limbs.

ANA—antinuclear antibodies; BMD—bone mineral density; BMI—body mass index; DLCO—diffusive capacity of carbon monoxide;DLCO/VA—diffusive capacity of carbon monoxide divided by alveolar volume; DXA—dual-energy X-ray absorptiometry; ENA—extractable nuclear antigen;ESR—erythrocyte sedimentation rate; EWGSOP—European Working Group on Sarcopenia in Older People; HGS—hand grip strength; ILD—interstitial lung disease; NVC—nailfold videocapillaroscopy; RSMI—relative skeletal muscle index.

**Table 6:** Histopathological studies performed on SSc patients’ myocardium.

Author	Year	Subjects	Results
D’Angelo et al. [147]	1969	The study included 58 autopsy reports of SSc patients.	In 81% of the patients, myocardial fibrosis was present, with significant prevalence at younger ages. Only 17% had lesions on the small coronary arteries.
Bulkley et al. [148]	1976	The study included 52 patients with autopsy records. A total of 28 patients had systemic hypertension, 25 had congestive heart failure, and 7 had angina pectoris.	Areas of myocardial fibrosis were found in 26 patients. The intramural coronary arteries were normal in all patients. The fibrosis was equally distributed in the left and right ventricles and extended to the endocardium in the majority of the cases. A total of 13 patients had severe fibrosis, 10 had moderate to mild, and 24 had no myocardial fibrosis. Muscle cell necrosis and contraction band formation were found as a response to inflammation and fibrosis. Contraction band necrosis was present in 77% of the patients with severe fibrosis and in 8% of the patients without fibrosis. The myocardial lesions were independent of pulmonary, renal, or hypertensive disease.
Murata et al. [149]	1998	The study included 95 patients, of which 31 had dcSSc. During the follow-up period, 14 died and 6 patients underwent an autopsy.	From the autopsy results, patchy fibrosis was found in 4 patients. Left ventricular hypertrophy was found in 4 patients. Two patients died of cardiac causes, one with cardiogenic shock and one with acute myocardial infarction.
Fernandes et al. [150]	2003	The study included 19 patients, of which 10 had lcSSc. All patients did not have any cardiac symptoms.	The interstitial collagen volume was present in 94% of the SSc group, with no significant difference between the types of disease. Perivascular fibrosis was increased in the SSc group, but with no statistical significance.
Mueller et al. [151]	2014	The study included 25 patients, of which 18 had dcSSc. All patients had clinical signs of cardiac involvement and 20% had arterial hypertension.	Fibrosis was present in all myocardium biopsies. Inflammation was present in 24 patients, 10 of them having single inflammatory cells, 8 having a few foci of inflammation, 4 having several foci of inflammation, and 2 having pronounced inflammation. Severe fibrosis and hyperplasia of smooth muscle cells were described after immunohistological staining with smooth muscle actin. The cardiovascular event rate was 25% for those with few foci of inflammation and 50% for those with moderate to severe inflammation.
Sandmeier et al. [142]	2015	The study included 11 autopsy reports, 8 from dcSSc. Of these, 4 died of cardiovascular causes.	Myocardial fibrosis was described in 5 patients, of which 3 had also coronary arteriosclerosis and 4 had generalized atherosclerosis. Only 3 patients with myocardial fibrosis had conduction block or diastolic failure and only one had palpitations.
De Luca et al. [152]	2020	The study included 34 patients that underwent endomyocardial biopsy with proven myocarditis (12 with SSc and virus negative myocarditis, 12 with isolated virus negative myocarditis, and 10 with virus negative myocarditis related to other systemic autoimmune diseases).	Myocardial fibrosis was higher, with a higher fibrosis score in SSc-related VNM, compared with isolated VNM and VNM from other autoimmune diseases. The percentage of myocardial fibrosis correlated positively with the extent of skin fibrosis evaluated through modified Rodnan skin score. The results did not reach statistical significance between the dc/lcSSc groups and did not correlate with skeletal myositis, cardiac enzymes, or pulmonary function tests.

dcSSc—diffuse cutaneous form; lcSSc—limited cutaneous form; VNM—virus-negative myocarditis.

**Table 7:** Recent studies that used cardiac magnetic resonance to evaluate systemic sclerosis patients.

Author	Year	Subjects	CMR Results
Sano et al. [153]	2014	The study included 40 patients. Patients with a history of coronary arterial disease were excluded.	LGE was described in 17.5% of the patients, distributed mainly in the mid-myocardial wall as follows: 8 sub-epicardial, 13 mid-myocardial, and 5 sub-endocardial. The pattern of LGE was striated in 23 cases and patchy in 3 cases. LGE-positive patients were more frequently symptomatic (NYHA classes > II), but no correlation was made with disease duration, type, or antibodies. Patients with LGE also had low LVEF, LV asynergy, and low RVEF.
Rodríguez-Reyna et al. [154]	2015	There were 62 patients included, of which 29 had dcSSc. None of the subjects had traditional cardiovascular risk factors. Patients that had a cardiac disease before the onset of SSc were excluded.	Myocardial fibrosis was described in 45% of the cases, was associated with dcSSc form, did not follow the coronary artery territories, and was mostly in the mesocardium. The most common pattern is intramural fibrosis in bands, found in 36% of cases. In the left ventricle basal–septal anterior and inferior segments were more frequently involved. The LVEF had lower values in patients with myocardial fibrosis. A total of 79% of the patients had subendocardial perfusion defects, suggesting a possible microvascular origin of the cardiac involvement.
Mavrogeni et al. [155]	2015	The study included 50 asymptomatic SSc patients with dcSSc, but only 46 CMR were available. They were compared with 20 healthy controls. Patients that underwent CMR had no history or symptoms of cardiac disease.	Acute myocardial inflammation was described in 2 patients on T2 images and they developed clinical signs of acute myocarditis. Fibrosis was present in 40 patients, although no LGE was identified. In the SSc group a diffused scar pattern was defined and followed the distribution of coronary arteries. In 44 patients there was a severely reduced MPRI after a perfusion–fibrosis protocol, correlated with the presence of digital ulcers.
Barison et al. [156]	2015	The study included 30 SSc patients, of which 28 had lcSSC and 10 healthy controls. All patients were asymptomatic or paucisymptomatic.	LGE was described in 23% of subjects with a patchy pattern in 3 patients, subepicardial in 2 patients, mid-wall in one patient, and subendocardial/transmural in one patient. Myocardial ECV was higher compared with healthy controls, but with no difference between those with and without LGE. Myocardial ECV correlated with skeletal muscle ECV.
Mavrogeni et al. [157]	2016	105 patients were evaluated with dcSSc without any history of cardiac disease, but with atypical cardiac symptoms.	A total of 25 patients had Q waves in V1-V6 on ECG. Of these, 24 had a patchy intramyocardial LGE pattern involving mostly the intraventricular septum. Eight patients had Q waves in II, III, and aVF and all of them had patchy intramyocardial LGE pattern in the inferior wall of LV. Five patients had Q waves in V1-V5, II, III, and aVF, in which cases a patchy intramyocardial LGE pattern was present in the anterior and inferolateral wall of LV.
Krumm et al. [141]	2016	The study included 20 patients, of which 19 had dcSSc. The participants underwent an endomyocardial biopsy with results previously reported by Mueller et al. [151].	LGE was present in 3 out of 19 patients with intermediate signal intensity in linear and patchy patterns. In another 10 patients, the LGE pattern was diffuse. In order to have myocardial involvement, CMR pathologic findings should be present in at least 3 of the following: pericardial effusion, pathologic LV/RV contractility, reduced LVEF or RVEF, positive LGE and RV dilatation. Positive CMR results were found in 7 patients with three categories, 9 with four categories, and 4 with five categories.
Kobayashi et al. [158]	2016	The 15 patients with SSc were evaluated, of which 8 had lcSSc. None of the patients had cardiac specific symptomatology. The study also included 10 healthy controls.	Non-segmental perfusion defects were present in 7 patients and associated with digital ulcers, while LGE was observed in another 4. The study showed that in individuals with no clinical signs of cardiovascular disease there is a decreased regional function that might predict other myocardial abnormalities.
Meduri et al. [159]	2017	The study included 50 patients, 19 with lcSSc and a mean disease duration of 73 ± 70.2 months. All of the participants had a form of cardiac involvement.	A pathologic MRI was found in 40 patients. Myocardial edema was described on T2-STIR images in 5 patients indicating myocardial injury or myocarditis. Increased interventricular septum thickness was present in 2 patients. Delayed enhancement was detected in 17 patients with 3 patterns, intramural, subepicardial and subendocardial, but with no difference between disease type.
Mavrogeni et al. [160]	2017	The study evaluated 72 patients, of which 62 had dcSSc. Patients with heart impairment were excluded.	Despite the lack of cardiac symptoms, 7 dcSSc and 2 lcSSc patients had myocarditis according to CMR. No correlation was found between CMR findings and blood inflammatory indices, cTnT or disease type. After treatment with prednisone and azathioprine, at 6-month follow-up no signs of myocarditis were present on CMR.
Hromadka et al. [161]	2017	The study included 33 SSc patients (87.9% with dcSSc) and 20 healthy controls.	In SSc group, there was a higher prevalence of LGE in the form of small focal areas with non-ischemic patterns in the intra-myocardial layer. Myocardial edema was present in one patient on T2 STIR sequence. GDF 15 had a significantly higher concentration and was correlated with ECV and native T1 in SSc patients. CMR fibrosis findings were correlated with serum galectin-3 levels, ECV and native T1.
Giacomelli et al. [162]	2017	The study included 16 SSc patients at recent onset, immediately after diagnosis and with no signs of internal organ involvement.	Perfusion defects were described in 16 patients, in five cases being sub-endocardial unrelated to coronary artery blood flow. None of these patients were LGE-positive. Only in one patient was the perfusion defect in the basal inferoseptal segment present during the stress and rest phase and associated with a positive LGE, suggesting a fibrotic lesion.
Muresan et al. [163]	2018	There were 30 patients included, of which 16 had dcSSc. Patients with a history of ischemic heart disease or known myocarditis were excluded.	Myocardial fibrosis was described in 83.3% of the cases, being more prevalent in the dcSSc type. There were no significant differences in the prevalence and characteristics of myocardial fibrosis between patients with dcSSc and lcSSc, apart from a higher prevalence of subepicardial fibrosis in the dcSSc and a higher prevalence of midwall fibrosis in the lcSSc. A total of 60% of the patients with myocardial fibrosis had ventricular arrhythmias or conduction disorders. Myocardial fibrosis was present with no difference between those with or without arrhythmias or conduction disorders.
Lee et al. [164]	2018	The study included 24 SSc patients, 13 with dcSSc, with early disease and 12 healthy controls. Of these, 67% had cardiac symptoms and 50% had hypertension.	LGE was described in 33% of patients with SSc compared with 0% in healthy controls. In the other 16 patients without LGE, diffuse myocardial fibrosis was found on T1 mapping techniques, ECV being the best index. They also found a positive correlation between mRSS and T1 mapping indexes.
Gyllenhammar et al. [165]	2018	The study evaluated 19 SSc patients (7 with dcSSc and 12 with lcSSc) and 22 healthy controls. No patient had a history of ischemic heart disease.	Global myocardial perfusion was similar in the controls and SSc group at rest, but during adenosine infusion SSc patients had lower myocardial perfusion without connection to the disease type. Hypoperfusion at stress can be considered a possible marker for cardiac disease in SSc. In 3 patients LGE was present with septal fibrosis in the right ventricular insertion points.
Bissell et al. [166]	2018	The study included 19 patients with ILR inserted, of which 32% suffered from dcSSc. No patient had a history of systemic hypertension. A total of 15 of these patients underwent CMR with LGE and ECV data available only for 14 of them.	Five of fourteen patients had LGE as evidence of focal fibrosis. These patients had higher levels of cardiac enzymes (hs-TnI and NT-proBNP). Higher ECV values were identified in patients with significant arrhythmias and were also correlated with higher levels of hs-Tnl.
Tipparot et al. [167]	2019	The study included 30 patients with SSc, 73.3% with dcSSc with a median duration of disease of 2 years.	Myocardial inflammation was described in 73.3% of cases, more often in women and dcSSc. Patients with myocardial inflammation were younger at onset, with higher mRSS at onset, and more frequently had a FVC < 70% and hand deformity. No correlation was found between inflammatory markers and cardiac enzymes.
Sugiyama et al. [168]	2019	The study included 49 patients, of which 25 had dcSSc.	Morphological myocardial changes were present in 29% of the patients, 55% of patients having an asymptomatic form. LGE was described in 55% of the patients, mostly in a linear pattern without coronary artery distribution and more frequently in patients with morphological changes. LGE presence was correlated with anti-Scl-70 positivity and higher BNP levels.
Rodríguez-Reyna et al. [169]	2019	The study followed the subjects previously described by Rodríguez-Reyna et al. in 2015.	After 43.5 months of follow-up, heart failure and coronary artery disease were more frequently found in patients with myocardial fibrosis, while digital ulcers were associated with microvascular damage. Myocardial fibrosis of the middle LV segments was found to be an independent predictor of heart failure, together with elevated ultrasensitive CRP and higher mRSS.
Markousis-Mavrogenis et al. [170]	2019	The study evaluated 50 patients with dcSSc, of which 31 were included in the event group as they experienced one or more cardiac events 3–10 days before recruitment.	In the beginning of the study, only 29% of the event group had elevated hs-cTnT values and cardiac functional impairment. The percentage increased to 40% in the event group and to 10 % in the no-event group after a median follow-up of 1.2 years. In the event group, the LGE values were higher compared with the other group, but with no difference between the T2 mapping or native T1 mapping. The only variables that could separate the event and no-event groups at baseline and independently predict prognosis at follow-up were LGE, T2 mapping, and native T1 mapping.
Gigante et al. [171]	2019	The study evaluated 20 patients with SSc and 10 healthy controls. Patients with cardiac involvement were excluded.	Focal areas of LGE were present in 5 patients, with patchy mesocardial distribution in the basal lateral wall or basal inferior septum, similar to a cardiac Raynaud’s phenomenon. Myocardial blood flow was similar in the two groups.
Bratis et al. [172]	2019	The research included 54 SSc patients (30 of them with lcSSc) with no known clinical cardiac symptoms and 21 healthy controls. Only 47 of the 54 patients underwent CMR.	LGE pattern consistent with myocardial infarction was present in 4 patients, and fibrosis at right ventricle insertion was detected in 9 patients. In the other 37 patients, no localized fibrosis was found. When compared with individuals without fibrosis, LV longitudinal strain was lower in patients with insertion fibrosis and significantly lower in patients with infarction.
Terrier et al. [173]	2020	The study included 40 patients, 19 with dcSSc. Four patients had a form of myocardial involvement and seven had systemic hypertension.	T1 values were higher in SSc patients, with higher quartiles being associated with dcSSc form, suggesting an increased collagen myocardial infiltration. Patients with higher T1 values had a more severe phenotype, with extensive skin involvement and ILD, low use of ACEi and vasodilators, and usage of glucocorticoids. Increased T1 values were also associated with microvascular myocardial impairment and a higher incidence of arrhythmogenic events. LGE was described in 5 patients, especially intramural and subepicardial, without correlation with the disease type. One patient had myocardial edema.
Poindron et al. [174]	2020	There were 72 patients included, of which 38 had dcSSc, 21 had a disease duration < 2 years, 19 had hypertension, 7 had ischemic heart disease and 8 peripheral artery disease.	On native T1 mapping, 50% of the patients showed elevated T1, indicating a significant prevalence of diffuse myocardial fibrosis. Focal fibrosis on LGE was present in 25% of the patients and was associated with decreased LVEF < 50%, although no correlation was found between myocardial LGE and T1 mapping values. In the elevated T1 group, 13 patients had a normal echocardiography with no clinical sign of heart failure.
Mavrogeni et al. [175]	2020	The study subjects consisted of 150 participants, of which 79 had cardiovascular symptoms at inclusion and 89 had dcSSc.	A total of 48.7% of the SAnCtUS cohort had one or more rhythm disturbances. LGE was identified as a predictor of supraventricular rhythm disturbance after multiple comparisons. T2 ratio and LGE are also predictors of any type of rhythm disturbances. This proves an important relationship between inflammation or fibrosis and rhythm disturbances.
Markousis-Mavrogenis et al. [176]	2020	The study evaluated 59 patients with dcSSc and cardiac symptomatology, 34 with a clinical suspicion of infectious myocarditis, and 31 healthy controls.	LV mass and T2 signal ratio were significantly higher in myocarditis patients compared with SSc. However, T1 mapping and EVC were lower in the myocarditis group compared with the SSc group. Furthermore, only 25% of SSc symptomatic patients had an acute inflammatory process, whereas all myocarditis patients had at least one pathologic T2-based index.
Galea et al. [177]	2020	The study evaluated 40 SSc patients with a disease duration of 8.7 ± 7.3 years without cardiac symptoms and 10 healthy controls.	Areas of focal edema were present in 12% of patients on T2 imaging. Focal areas of LGE were also present in 30% of the subjects, with a nonischemic pattern, mostly patchy mesocardial distribution in the basal lateral wall or basal inferior septum. SSc patients had higher native T1 (72%) and T2 (52%) values and ECV (52%) compared with healthy controls, even if there was no ventricular contractile dysfunction. Almost 60% of lcSSc and 44% of dcSSc patients had a reduced increase in myocardial blood flow after cold pressor test, suggesting a reduced vasodilatory response as a consequence of microvascular dysfunction.
De Luca et al. [152]	2020	The study included 34 patients (12 with SSc and virus-negative myocarditis, 12 with isolated virus-negative myocarditis, and 10 with virus-negative myocarditis related to other systemic autoimmune diseases). Half of the SSc group subjects had early disease (<3 years).	Myocardial edema was more frequently found in isolated virus-negative myocarditis and virus-negative myocarditis associated with other systemic autoimmune diseases. LGE was present in all SSc patients with virus-negative myocarditis and in more than 80% of the other two groups predominantly subepicardial with midwall/patchy patterns. No correlation was found between myocardial fibrosis on biopsy and CMR results.
Bordonaro et al. [178]	2020	The study comprised 33 individuals with SSc and 33 healthy controls. Twenty of the thirty-three patients suffered from dcSSc. Twelve patients (36.4%) in the patient group showed no clinical cardiac symptoms.	Compared with the healthy group, T1 and T2 mappings were noticeably greater, with the native T1 value increased for 16 patients. A total of 18 patients had significantly higher ECV compared with the healthy controls.LGE was seen in eight patients, while none of the healthy volunteers had cardiac LGE. LGE, higher native T1, and higher ECV were predictors of adverse clinical outcomes and could represent novel markers of risk stratification in SSc.
Hromadka et al. [179]	2021	The study included 25 patients out of 33 that were previously described by Hromadka et al. in 2017 [161].	Although there are some individuals who have CMR indicators of the progression or regression of cardiac degeneration, the mean values of native T1 time and ECV did not change significantly during a 5-year follow-up. The GDF-15 showed a strong correlation with the change in clinical scores for the disease’s progression in the skin or lungs, becoming a potential biomarker for disease activity.
Dumitru et al. [180]	2021	The study included 83 SSc patients with a disease duration of 7 years. A total of 22 patients had at least one cardiovascular risk factor associated.	Focal LGE fibrosis was described in 21% of the SSc patients and in none of the healthy controls in a non-ischemic pattern, with a linear pattern in 9 subjects, focal pattern in 6 subjects, and diffuse pattern in 2 subjects. The LGE distribution was mainly midwall and subepicardial. Furthermore, it was associated with a higher mRSS score, higher CRP, and higher hs-TnI levels. ECV was higher in 52% of SSc patients compared with healthy controls and was associated with digital ulcers and higher values of NT-proBNP. Native T1 values were higher in 61% of SSc patients. No patient had regional perfusion defects suggestive of coronary artery disease. Treatment with DMARD or angiotensin-converting enzymes inhibitors was not associated with CMR results of fibrosis and vasculopathy.
Dumitru et al. [181]	2021	The study evaluated 74 SSc patients, including 50 with lcSSc and with no clinical SSc primary heart involvement or history of heart disease. In 10 patients there were cardiovascular events recorded during follow-up.	In 72 cases LGE was available and 14 patients had focal fibrosis in a noncoronary distribution. Of these, only one patient had a cardiovascular outcome. A total of 48 out of 71 patients had higher ECV. The probability of cardiovascular events was higher in patients with higher NT-proBNP, ECV, or hs-TnI values, but with no difference in patients with LGE compared with those without LGE.
Vos et al. [182]	2022	The study included 100 patients, half of them with dcSSc. A total of 54% presented with dyspnea and 24% with chest pain. CMR was performed for suspected myocarditis (41%), followed by newly onset heart failure (19%).	LGE was present in 21% of the patients, in 10 cases being a non-ischemic pattern. In these cases, the patients had a worse outcome compared with those without LGE.
Ross et al. [95]	2022	The study included 34 patients, with 31 CMI available for analysis. There were 19 patients with dcSSc.	Focal areas of fibrosis of LGE were present in 9 patients. More than 90% of the subjects presented high native T1 times. In 40% of the subjects, muscle edema was present on CMR. No association was found between diffuse myocardial inflammation and SSc-associated myopathy.
Palumbo et al. [183]	2022	The study included 31 patients, of which 10 had dcSSc, 10 had lcSSc, and 11 fulfilled the VEDOSS criteria for early SSc. They were followed up for 6.8 years and compared with a healthy group of 23 participants.	Tissue tracking on CMR enables the identification of early cardiac involvement in SSc when compared with healthy controls. Disease accrual damage predominately affected the dcSSc patients, even though there was no statistically significant difference between the three subgroups.
Knight et al. [184]	2022	The study evaluated 260 patients, 167 with lcSSc and a median time from SSc diagnosis of 9 years. A total of 30% of the participants had an overlap connective tissue disease and 43% had pulmonary hypertension. The main indication for CMR was pulmonary hypertension.	LGE was present in 37% of the subjects, mainly with major ventricular insertion point patterns. In the SSc and pulmonary hypertension group there was higher native myocardial T1 and T2 values, with higher myocardial ECV and a greater prevalence of major ventricular insertion point LGE and pericardial effusion. Native myocardial T1 values were an independent predictor of mortality. They defined 5 clusters based on CMR modifications that are associated with different outcomes.

ACEi—angiotensin-converting enzyme inhibitors; BNP—brain natriuretic peptide; CMR—cardiac magnetic resonance; CRP—C-reactive protein; dcSSc—diffuse cutaneous form of systemic sclerosis; DMARD—disease-modifying antirheumatic drugs; ECG—electrocardiography; ECV—mean extracellular volume; FVC—forced vital capacity; GDF-15—Growth/Differentiation Factor-15; hs-cTnT—high-sensitivity cardiac troponin T; hs-TnI—high-sensitivity troponin I; ILD—interstitial lung disease; ILR—implantable loop recorder; lcSSc—limited cutaneous form of systemic sclerosis; LGE—late gadolinium enhancement; LV—left ventricle; LVEF—left ventricle ejection fraction; mRSS—modified Rodnan skin score; NT-proBNP—N-terminal prohormone of brain natriuretic peptide; NYHA—New York Heart Association Classification; RV—right ventricle; RVEF—right ventricle ejection fraction; STIR—Short Tau Inversion Recovery.

## Data Availability

Not applicable.

## References

[1] Denton C., Khanna D. (2017). Systemic sclerosis. Lancet.

[2] Varga J., Trojanowska M., Kuwana M. (2017). Pathogenesis of systemic sclerosis: Recent insights of molecular and cellular mechanisms and therapeutic opportunities. J. Scleroderma Relat. Disord..

[3] Ferreli C., Gasparini G., Parodi A., Cozzani E., Rongioletti F., Atzori L. (2017). Cutaneous Manifestations of Scleroderma and Scleroderma-Like Disorders: A Comprehensive Review. Clin. Rev. Allergy Immunol..

[4] Di Battista M., Barsotti S., Orlandi M., Lepri G., Codullo V., Della Rossa A., Guiducci S., Del Galdo F. (2021). One year in review 2021: Systemic sclerosis. Clin. Exp. Rheumatol..

[5] Cutolo M., Soldano S., Smith V. (2019). Pathophysiology of systemic sclerosis: Current understanding and new insights. Expert Rev. Clin. Immunol..

[6] Asano Y. (2020). The Pathogenesis of Systemic Sclerosis: An Understanding Based on a Common Pathologic Cascade across Multiple Organs and Additional Organ-Specific Pathologies. J. Clin. Med..

[7] Korman B. (2019). Evolving insights into the cellular and molecular pathogenesis of fibrosis in systemic sclerosis. Transl. Res..

[8] Truchetet M.E., Brembilla N.C., Chizzolini C. (2021). Current Concepts on the Pathogenesis of Systemic Sclerosis. Clin. Rev. Allergy Immunol..

[9] Romano E., Rosa I., Fioretto B., Matucci-Cerinic M., Manetti M. (2021). New Insights into Profibrotic Myofibroblast Formation in Systemic Sclerosis: When the Vascular Wall Becomes the Enemy. Life.

[10] Trojanowska M. (2010). Cellular and molecular aspects of vascular dysfunction in systemic sclerosis. Nat. Rev. Rheumatol..

[11] Sallam H., McNearney T.A., Chen J.D.Z. (2006). Systematic review: Pathophysiology and management of gastrointestinal dysmotility in systemic sclerosis (scleroderma). Aliment. Pharmacol. Ther..

[12] Romano E., Rosa I., Fioretto B.S., Matucci-Cerinic M., Manetti M. (2022). The Role of Pro-fibrotic Myofibroblasts in Systemic Sclerosis: From Origin to Therapeutic Targeting. Curr. Mol. Med..

[13] Lóránd V., Czirják L., Minier T. (2014). Musculoskeletal involvement in systemic sclerosis. Presse. Med..

[14] Randone S.B., Guiducci S., Cerinic M.M. (2008). Musculoskeletal involvement in systemic sclerosis. Best Pract. Res. Clin. Rheumatol..

[15] Corallo C., Cutolo M., Volpi N., Franci D., Aglianó M., Montella A., Chirico C., Gonnelli S., Nuti R., Giordano N. (2016). Histopathological findings in systemic sclerosis-related myopathy: Fibrosis and microangiopathy with lack of cellular inflammation. Ther. Adv. Musculoskelet. Dis..

[16] Ranque B., Authier F., Berezne A., Guillevin L., Mouthon L. (2007). Systemic Sclerosis-Associated Myopathy. Ann. N. Y. Acad. Sci..

[17] Jinnin M. (2020). ‘Narrow-sense’ and ‘broad-sense’ vascular abnormalities of systemic sclerosis. Immunol. Med..

[18] Maciejewska M., Sikora M., Maciejewski C., Alda-Malicka R., Czuwara J., Rudnicka L. (2022). Raynaud’s Phenomenon with Focus on Systemic Sclerosis. J. Clin. Med..

[19] Bruni C., Cuomo G., Rossi F.W., Praino E., Bellando-Randone S. (2018). Kidney involvement in systemic sclerosis: From pathogenesis to treatment. J. Scleroderma Relat. Disord..

[20] van Caam A., Vonk M., van den Hoogen F., van Lent P., van der Kraan P. (2018). Unraveling SSc Pathophysiology; The Myofibroblast. Front. Immunol..

[21] Manetti M., Romano E., Rosa I., Guiducci S., Bellando-Randone S., De Paulis A., Ibba-Manneschi L., Matucci-Cerinic M. (2017). Endothelial-to-mesenchymal transition contributes to endothelial dysfunction and dermal fibrosis in systemic sclerosis. Ann. Rheum. Dis..

[22] Di Benedetto P., Ruscitti P., Berardicurti O., Vomero M., Navarini L., Dolo V., Cipriani P., Giacomelli R. (2021). Endothelial-to-mesenchymal transition in systemic sclerosis. Clin. Exp. Immunol..

[23] Rosa I., Romano E., Fioretto B.S., Manetti M. (2020). The contribution of mesenchymal transitions to the pathogenesis of systemic sclerosis. Eur. J. Rheumatol..

[24] Manetti M., Neumann E., Milia A.F., Tarner I.H., Bechi P., Matucci-Cerinic M., Ibba-Manneschi L., Müller-Ladner U. (2007). Severe fibrosis and increased expression of fibrogenic cytokines in the gastric wall of systemic sclerosis patients. Arthritis Care Res..

[25] Jimenez S.A., Piera-Velazquez S. (2016). Endothelial to mesenchymal transition (EndoMT) in the pathogenesis of Systemic Sclerosis-associated pulmonary fibrosis and pulmonary arterial hypertension. Myth or reality?. Matrix Biol..

[26] Wang G., Jacquet L., Karamariti E., Xu Q. (2015). Origin and differentiation of vascular smooth muscle cells. J. Physiol..

[27] Shi J., Yang Y., Cheng A., Xu G., He F. (2020). Metabolism of vascular smooth muscle cells in vascular diseases. Am. J. Physiol. Circ. Physiol..

[28] Lechartier B., Berrebeh N., Huertas A., Humbert M., Guignabert C., Tu L. (2022). Phenotypic Diversity of Vascular Smooth Muscle Cells in Pulmonary Arterial Hypertension: Implications for Therapy. Chest.

[29] Miano J.M., Fisher E.A., Majesky M.W. (2021). Fate and State of Vascular Smooth Muscle Cells in Atherosclerosis. Circulation.

[30] Sorokin V., Vickneson K., Kofidis T., Woo C.C., Lin X.Y., Foo R., Shanahan C.M. (2020). Role of Vascular Smooth Muscle Cell Plasticity and Interactions in Vessel Wall Inflammation. Front. Immunol..

[31] Altorok N., Nada S., Kahaleh B. (2016). The isolation and characterization of systemic sclerosis vascular smooth muscle cells: Enhanced proliferation and apoptosis resistance. J. Scleroderma Relat. Disord..

[32] Bălănescu P., Bălănescu E., Bălănescu A. (2017). IL-17 and Th17 cells in systemic sclerosis: A comprehensive review. Romanian J. Intern. Med..

[33] Liu M., Yang J., Xing X., Cui X., Li M. (2014). Interleukin-17A promotes functional activation of systemic sclerosis patient-derived dermal vascular smooth muscle cells by extracellular-regulated protein kinases signalling pathway. Arthritis Res. Ther..

[34] Liu M., Yang J., Li M. (2015). Tanshinone IIA attenuates interleukin-17A-induced systemic sclerosis patient-derived dermal vascular smooth muscle cell activation via inhibition of the extracellular signal-regulated kinase signaling pathway. Clinics.

[35] Karatas A., Celik C., Oz B., Akar Z.A., Etem E.O., Dagli A.F., Koca S.S. (2021). Secukinumab and metformin ameliorate dermal fibrosis by decreasing tissue interleukin-17 levels in bleomycin-induced dermal fibrosis. Int. J. Rheum. Dis..

[36] Hegner B., Schaub T., Catar R., Kusch A., Wagner P., Essin K., Lange C., Riemekasten G., Dragun D. (2016). Intrinsic Deregulation of Vascular Smooth Muscle and Myofibroblast Differentiation in Mesenchymal Stromal Cells from Patients with Systemic Sclerosis. PLoS ONE.

[37] Jiang Y., Turk M.A., Pope J.E. (2020). Factors associated with pulmonary arterial hypertension (PAH) in systemic sclerosis (SSc). Autoimmun. Rev..

[38] Trojanowska M. (2017). Pulmonary Hypertension Associated With Scleroderma and Connective Tissue Disease: Potential Molecular and Cellular Targets. Adv. Pulm. Hypertens..

[39] Kherbeck N., Tamby M.C., Bussone G., Dib H., Perros F., Humbert M., Mouthon L. (2011). The Role of Inflammation and Autoimmunity in the Pathophysiology of Pulmonary Arterial Hypertension. Clin. Rev. Allergy Immunol..

[40] Boin F., Erre G.L., Posadino A.M., Cossu A., Giordo R., Spinetti G., Passiu G., Emanueli C., Pintus G. (2014). Oxidative stress-dependent activation of collagen synthesis is induced in human pulmonary smooth muscle cells by sera from patients with scleroderma-associated pulmonary hypertension. Orphanet J. Rare Dis..

[41] Svegliati S., Amico D., Spadoni T., Fischetti C., Finke D., Moroncini G., Paolini C., Tonnini C., Grieco A., Rovinelli M. (2017). Agonistic Anti-PDGF Receptor Autoantibodies from Patients with Systemic Sclerosis Impact Human Pulmonary Artery Smooth Muscle Cells Function In Vitro. Front. Immunol..

[42] Liang M., Lv J., Jiang Z., He H., Chen C., Xiong Y., Zhu X., Xue Y., Yu Y., Yang S. (2020). Promotion of Myofibroblast Differentiation and Tissue Fibrosis by the Leukotriene B4-Leukotriene B4 Receptor Axis in Systemic Sclerosis. Arthritis Rheumatol..

[43] Qian J., Tian W., Jiang X., Tamosiuniene R., Sung Y.K., Shuffle E.M., Tu A.B., Valenzuela A., Jiang S., Zamanian R.T. (2015). LTB4 activates pulmonary artery adventitial fibroblasts in pulmonary hypertension. Hypertension.

[44] Noda S., Asano Y., Nishimura S., Taniguchi T., Fujiu K., Manabe I., Nakamura K., Yamashita T., Saigusa R., Akamata K. (2014). Simultaneous downregulation of KLF5 and Fli1 is a key feature underlying systemic sclerosis. Nat. Commun..

[45] Shu T., Xing Y., Wang J. (2021). Autoimmunity in Pulmonary Arterial Hypertension: Evidence for Local Immunoglobulin Production. Front. Cardiovasc. Med..

[46] Huertas A., Tu L., Humbert M., Guignabert C. (2020). Chronic inflammation within the vascular wall in pulmonary arterial hypertension: More than a spectator. Cardiovasc. Res..

[47] Ntokou A., Dave J.M., Kauffman A.C., Sauler M., Ryu C., Hwa J., Herzog E.L., Singh I., Saltzman W.M., Greif D.M. (2021). Macrophage-derived PDGF-B induces muscularization in murine and human pulmonary hypertension. JCI Insight.

[48] Trojanowska M. (2008). Role of PDGF in fibrotic diseases and systemic sclerosis. Rheumatology.

[49] Baroni S.S., Santillo M., Bevilacqua F., Luchetti M., Spadoni T., Mancini M., Fraticelli P., Sambo P., Funaro A., Kazlauskas A. (2006). Stimulatory Autoantibodies to the PDGF Receptor in Systemic Sclerosis. N. Engl. J. Med..

[50] Atanelishvili I., Akter T., Noguchi A., Vuyiv O., Wollin L., Silver R.M., Bogatkevich G.S. (2019). Antifibrotic efficacy of nintedanib in a cellular model of systemic sclerosis-associated interstitial lung disease. Clin. Exp. Rheumatol..

[51] Arts M.R., Baron M., Chokr N., Fritzler M.J., Servant M.J., the Canadian Scleroderma Research Group (CSRG) (2014). Systemic Sclerosis Immunoglobulin Induces Growth and a Pro-Fibrotic State in Vascular Smooth Muscle Cells through the Epidermal Growth Factor Receptor. PLoS ONE.

[52] Bussone G., Tamby M.C., Calzas C., Kherbeck N., Sahbatou Y., Sanson C., Ghazal K., Dib H., Weksler B.B., Broussard C. (2011). IgG from patients with pulmonary arterial hypertension and/or systemic sclerosis binds to vascular smooth muscle cells and induces cell contraction. Ann. Rheum. Dis..

[53] Petcu A., Ghib L.J., Grad S.M., Popovici C., Rogojan L., Rednic N.V., Rednic S. (2019). Upper gastrointestinal involvement in systemic sclerosis: Findings in a real-life setting. Exp. Ther. Med..

[54] Frech T.M., Mar D. (2018). Gastrointestinal and Hepatic Disease in Systemic Sclerosis. Rheum. Dis. Clin. N. Am..

[55] Voulgaris T., Karamanolis G. (2021). Esophageal manifestations in patients with scleroderma. World J. Clin. Cases.

[56] Denaxas K., Ladas D., Karamanolis P. (2018). Evaluation and management of esophageal manifestations in systemic sclerosis. Ann. Gastroenterol..

[57] Vischio J., Saeed F., Karimeddini M., Mubashir A., Feinn R., Caldito G., Striegel K., Rothfield N. (2012). Progression of Esophageal Dysmotility in Systemic Sclerosis. J. Rheumatol..

[58] Lepri G., Guiducci S., Bellando-Randone S., Giani I., Bruni C., Blagojevic J., Carnesecchi G., Radicati A., Pucciani F., Marco M.-C. (2013). Evidence for oesophageal and anorectal involvement in very early systemic sclerosis (VEDOSS): Report from a single VEDOSS/EUSTAR centre. Ann. Rheum. Dis..

[59] Thonhofer R., Siegel C., Trummer M., Graninger W. (2010). Early endoscopy in systemic sclerosis without gastrointestinal symptoms. Rheumatol. Int..

[60] Kimmel J.N., Carlson D.A., Hinchcliff M., Carns M.A., Aren K.A., Lee J., Pandolfino J.E. (2016). The association between systemic sclerosis disease manifestations and esophageal high-resolution manometry parameters. Neurogastroenterol. Motil..

[61] Li B., Yan J., Pu J., Tang J., Xu S., Wang X. (2021). Esophageal Dysfunction in Systemic Sclerosis: An Update. Rheumatol. Ther..

[62] Roberts C., Hummers L., Ravich W., Wigley F., Hutchins G. (2006). A case-control study of the pathology of oesophageal diseae in systemic sclerosis (Scleroderma). Gut.

[63] Zuber-Jerger I., Müller A., Kullmann F., Gelbmann C.M., Endlicher E., Müller-Ladner U., Fleck M. (2009). Gastrointestinal manifestation of systemic sclerosis—Thickening of the upper gastrointestinal wall detected by endoscopic ultrasound is a valid sign. Rheumatology.

[64] Taroni J.N., Martyanov V., Huang C.-C., Mahoney J.M., Hirano I., Shetuni B., Yang G.-Y., Brenner D., Jung B., Wood T.A. (2015). Molecular characterization of systemic sclerosis esophageal pathology identifies inflammatory and proliferative signatures. Arthritis Res. Ther..

[65] Mcmahan Z.H., Hummers L.K. (2018). Gastrointestinal involvement in systemic sclerosis: Diagnosis and management. Curr. Opin. Rheumatol..

[66] Marie I., Levesque H., Ducrotté P., Denis P., Hellot M.F., Benichou J., Cailleux N., Courtois H. (2001). Gastric Involvement in Systemic Sclerosis: A Prospective Study. Am. J. Gastroenterol..

[67] Miller J., Gandhi N., Clarke J., McMahan Z. (2018). Gastrointestinal involvement in systemic sclerosis: An update. J. Clin. Rheumatol..

[68] Ibba-Manneschi L., Del Rosso A., Pacini S., Tani A., Bechi P., Cerinic M.M. (2002). Ultrastructural study of the muscle coat of the gastric wall in a case of systemic sclerosis. Ann. Rheum. Dis..

[69] Manetti M., Milia A.F., Benelli G., Messerini L., Matucci-Cerinic M., Ibba-Manneschi L. (2010). The gastric wall in systemic sclerosis patients: A morphological study. Ital. J. Anat. Embryol..

[70] Kumar S., Singh J., Rattan S., Dimarino A.J., Cohen S., Jimenez S.A. (2017). Review article: Pathogenesis and clinical manifestations of gastrointestinal involvement in systemic sclerosis. Aliment. Pharmacol. Ther..

[71] Sakkas L.I., Simopoulou T., Daoussis D., Liossis S.-N., Potamianos S. (2018). Intestinal Involvement in Systemic Sclerosis: A Clinical Review. Am. J. Dig. Dis..

[72] Brandler J.B., Sweetser S., Khoshbin K., Babameto M., Prokop L.J., Camilleri M. (2019). Colonic Manifestations and Complications Are Relatively Under-Reported in Systemic Sclerosis: A Systematic Review. Am. J. Gastroenterol..

[73] Polkowska-Pruszyńska B., Gerkowicz A., Szczepanik-Kułak P., Krasowska D. (2018). Small intestinal bacterial overgrowth in systemic sclerosis: A review of the literature. Arch. Dermatol. Res..

[74] Gregersen H., Liao D., Pedersen J., Drewes A. (2007). A new method for evaluation of intestinal muscle contraction properties: Studies in normal subjects and in patients with systemic sclerosis. Neurogastroenterol. Motil..

[75] Thoua N.M., Derrett-Smith E.C., Khan K., Dooley A., Shi-Wen X., Denton C.P. (2012). Gut fibrosis with altered colonic contractility in a mouse model of scleroderma. Rheumatology.

[76] Braber-Ymker M.D., Vonk M.C., Grünberg K., Lammens M., Nagtegaal I.D. (2020). Intestinal hypomotility in systemic sclerosis: A histological study into the sequence of events. Clin. Rheumatol..

[77] Pinto R.A., Corrêa Neto I.J.F., Nahas S.C., Bustamante Lopes L.A., Sobrado Júnior C.W., Cecconello I. (2018). Functional and Anatomical analysis of the anorectum of female scleroderma patients at a center for pelvic floor disorders. Arq. Gastroenterol..

[78] Sattar B., Chokshi R.V. (2019). Colonic and Anorectal Manifestations of Systemic Sclerosis. Curr. Gastroenterol. Rep..

[79] Follansbee W.P., Zerbe T.R., Medsger T.A. (1993). Cardiac and skeletal muscle disease in systemic sclerosis (scleroderma): A high risk association. Am. Heart J..

[80] Paik J., Mammen A., Wigley F., Gelber A. (2014). Myopathy in scleroderma, its identification, prevalence, and treatment: Lessons learned from cohort studies. Curr. Opin. Rheumatol..

[81] Jablonska S., Blaszcyk M. (1998). Scleromyositis: A scleroderma/polymyositis overlap syndrome. Clin. Rheumatol..

[82] Justo A.C., Guimarães F.S., Ferreira A.S., Soares M.S., Bunn P.S., Lopes A.J. (2017). Muscle function in women with systemic sclerosis: Association with fatigue and general physical function. Clin. Biomech..

[83] Pettersson H., Boström C., Bringby F., Walle-Hansen R., Jacobsson L., Svenungsson E., Nordin A., Alexanderson H. (2019). Muscle endurance, strength, and active range of motion in patients with different subphenotypes in systemic sclerosis: A cross-sectional cohort study. Scand. J. Rheumatol..

[84] Maundrell A., Proudman S., Limaye V. (2019). Prevalence of other connective tissue diseases in idiopathic inflammatory myopathies. Rheumatol. Int..

[85] Paik J. (2016). Myopathy in scleroderma and in other connective tissue diaseses. Curr. Opin. Rheumatol..

[86] Aguila L.A., da Silva H.C., Medeiros-Ribeiro A.C., Bunjes B.G., Luppino-Assad A.P., Sampaio-Barros P.D. (2020). Is exposure to environmental factors associated with a characteristic clinical and laboratory profile in systemic sclerosis? A retrospective analysis. Rheumatol. Int..

[87] Paik J.J. (2018). Muscle disease in scleroderma. Curr. Opin. Rheumatol..

[88] Walker U.A., Clements P.J., Allanore Y., Distler O., Oddis C.V., Khanna D., Furst D.E. (2017). Muscle involvement in systemic sclerosis: Points to consider in clinical trials. Rheumatology.

[89] Jung M., Bonner A., Hudson M., Baron M., Pope J., the Canadian Scleroderma Research Group (CSRG) (2013). Myopathy is a poor prognostic feature in systemic sclerosis: Results from the Canadian Scleroderma Research Group (CSRG) cohort. Scand. J. Rheumatol..

[90] Paik J.J., Wigley F.M., Mejia A.F., Hummers L.K. (2016). Independent Association of Severity of Muscle Weakness With Disability as Measured by the Health Assessment Questionnaire Disability Index in Scleroderma. Arthritis Care Res..

[91] Ranque B., Bérezné A., Le-Guern V., Pagnoux C., Allanore Y., Launay D., Hachulla E., Authier F.J., Gherardi R., Kahan A. (2010). Myopathies related to systemic sclerosis: A case-control study of associated clinical and immunological features. Scand. J. Rheumatol..

[92] Araujo C., Miossi R., De Souza F., Costa M., Da Silva A., Campos E., Zanoteli E., Shinjo S. (2021). Brachio-cervical inflammatory myopathy associated with systemic sclerosis. Case series and review of literature. Reumatismo.

[93] Shimada T., Higashida-Konishi M., Akiyama M., Hama S., Takei H., Izumi K., Oshima H., Okano Y. (2021). Dropped head in systemic sclerosis: A case based review. Rheumatol. Int..

[94] Tolédano C., Gain M., Kettaneh A., Baudin B., Johanet C., Chérin P., Rivière S., Cabane J., Tiev K.P. (2012). Aldolase predicts subsequent myopathy occurrence in systemic sclerosis. Arthritis Res. Ther..

[95] Ross L., Lindqvist A., Costello B., Hansen D., Brown Z., Day J.A., Stevens W., Burns A., Perera W., Pianta M. (2022). Using magnetic resonance imaging to map the hidden burden of muscle involvement in systemic sclerosis. Arthritis Res. Ther..

[96] Dumitru R.B., Goodall A.F., Broadbent D.A., Del Galdo F., Tan A.L., Biglands J.D., Buch M.H. (2021). First pilot study of extracellular volume MRI measurement in peripheral muscle of systemic sclerosis patients suggests diffuse fibrosis. Rheumatology.

[97] Ranque B., Authier F.J., Le-Guern V., Pagnoux C., Berezne A., Allanore Y., Launay D., Hachulla E., Kahan A., Cabane J. (2008). A descriptive and prognostic study of systemic sclerosis-associated myopathies. Ann. Rheum. Dis..

[98] Schanz S., Henes J., Ulmer A., Kötter I., Fierlbeck G., Claussen C.D., Horger M. (2012). Magnetic resonance imaging findings in patients with systemic scleroderma and musculoskeletal symptoms. Eur. Radiol..

[99] Zhou M., Jiang L., Nie L., Chen T., Zhang T., Sun W., Sutikno J., Du Y., Xue J. (2020). Myopathy is a Risk Factor for Poor Prognosis of Patients with Systemic Sclerosis: A retrospective cohort study. Medicine.

[100] Baumberger R., Jordan S., Distler O., Pfister P.B., Maurer B. (2021). Diagnostic measures for patients with systemic sclerosis-associated myopathy. Clin. Exp. Rheumatol..

[101] Lefebvre F., Giannini M., Ellezam B., Leclair V., Troyanov Y., Hoa S., Bourré-Tessier J., Satoh M., Fritzler M.J., Senécal J.-L. (2021). Histopathological features of systemic sclerosis-associated myopathy: A scoping review. Autoimmun. Rev..

[102] Paik J.J., Wigley F.M., Shah A.A., Corse A.M., Casciola-Rosen L., Hummers L.K., Mammen A.L. (2017). Association of Fibrosing Myopathy in Systemic Sclerosis and Higher Mortality. Arthritis Care Res..

[103] Siegert E., Uruha A., Goebel H.-H., Preuße C., Casteleyn V., Kleefeld F., Alten R., Burmester G.R., Schneider U., Höppner J. (2021). Systemic sclerosis-associated myositis features minimal inflammation and characteristic capillary pathology. Acta Neuropathol..

[104] Paik J.J., Wigley F.M., Lloyd T.E., Corse A.M., Casciola-Rosen L., Shah A.A., Boin F., Hummers L.K., Mammen A.L. (2015). Spectrum of Muscle Histopathologic Findings in Forty-Two Scleroderma Patients With Weakness. Arthritis Care Res..

[105] Koschik R.W., Fertig N., Lucas M.R., Domsic R.T., Medsger T.A. (2022). Anti-PM-Scl antibody in patients with systemic sclerosis. Clin. Exp. Rheumatol..

[106] Wielosz E., Dryglewska M., Majdan M. (2021). The prevalence and significance of anti-PM/Scl antibodies in systemic sclerosis. Ann. Agric. Environ. Med..

[107] Mahler M., Raijmakers R. (2007). Novel aspects of autoantibodies to the PM/Scl complex: Clinical, genetic and diagnostic insights. Autoimmun. Rev..

[108] Hamaguchi Y., Takehara K. (2018). Anti-nuclear autoantibodies in systemic sclerosis: News and perspectives. J. Scleroderma Relat. Disord..

[109] De Lorenzo R., Pinal-Fernandez I., Huang W., Albayda J., Tiniakou E., Johnson C., Milisenda J.C., Casal-Dominguez M., Corse A.M., Danoff S.K. (2018). Muscular and extramuscular clinical features of patients with anti-PM/Scl autoantibodies. Neurology.

[110] Cozzani E., Muracchioli A., Murdaca G., Beccalli M., Caprioli S., Zentilin P., Ameri P., Grosso M., Russo R., Carmisciano L. (2021). Correlation Between Skin and Affected Organs in 52 Sclerodermic Patients Followed in a Diseases Management Team: Development of a Risk Prediction Model of Organ-Specific Complications. Front. Immunol..

[111] D’Aoust J., Hudson M., Tatibouet S., Wick J., Mahler M., Baron M., Fritzler M.J., the Canadian Scleroderma Research Group (2014). Clinical and Serologic Correlates of Anti-PM/Scl Antibodies in Systemic Sclerosis: A Multicenter Study of 763 Patients. Arthritis Rheumatol..

[112] Wodkowski M., Hudson M., Proudman S., Walker J., Stevens W., Nikpour M., Assassi S., Mayes M.D., Tatibouet S., Wang M. (2015). Clinical correlates of monospecific anti-PM75 and anti-PM100 antibodies in a tri-nation cohort of 1574 systemic sclerosis subjects. Autoimmunity.

[113] Leurs A., Dubucquoi S., Machuron F., Balden M., Renaud F., Rogeau S., Lopez B., Lambert M., Morell-Dubois S., Maillard H. (2021). Extended myositis-specific and -associated antibodies profile in systemic sclerosis: A cross-sectional study. Jt. Bone Spine.

[114] Lazzaroni M.-G., Marasco E., Campochiaro C., DeVries-Bouwstra J., Gonzalez-Perez M.-I., Rojas-Serrano J., Hachulla E., Zanatta E., Barsotti S., Furini F. (2021). The clinical phenotype of systemic sclerosis patients with anti-PM/Scl antibodies: Results from the EUSTAR cohort. Rheumatology.

[115] Arandia N.I., Espinosa G., Del Castillo A.G., Tolosa-Vilella C., Colunga-Argüelles D., Lledó G.M., Comet L.S., Ortego-Centeno N., Hito J.A.V., Rubio-Rivas M. (2022). Anti-Polymyositis/Scl Antibodies in Systemic Sclerosis: Clinical Associations in a Multicentric Spanish Cohort and Review of the Literature. J. Clin. Rheumatol..

[116] Fredi M., Cavazzana I., Franceschini F. (2018). The clinico-serological spectrum of overlap myositis. Curr. Opin. Rheumatol..

[117] Van den Hoogen F., Khanna D., Fransen J., Johnson S.R., Baron M., Tyndall A., Matucci-Cerinic M., Naden R.P., Medsger T.A., Carreira P.E. (2013). 2013 classification criteria for systemic sclerosis: An American college of rheumatology/European league against rheumatism collaborative initiative. Ann. Rheum. Dis..

[118] Bottai M., Tjärnlund A., Santoni G., Werth V.P., Pilkington C., de Visser M., Alfredsson L., Amato A.A., Barohn R.J., Liang M.H. (2017). EULAR/ACR classification criteria for adult and juvenile idiopathic inflammatory myopathies and their major subgroups: A methodology report. RMD Open.

[119] Leclair V., D’Aoust J., Gyger G., Landon-Cardinal O., Meyer A., O'Ferrall E., Karamchandani J., Massie R., Ellezam B., Satoh M. (2021). Autoantibody profiles delineate distinct subsets of scleromyositis. Rheumatology.

[120] Szabó K., Bodoki L., Nagy-Vincze M., Béldi T., Vincze A., Zilahi E., Varga J., Szűcs G., Dankó K., Griger Z. (2022). Clinical, Serological, and Genetic Characteristics of a Hungarian Myositis-Scleroderma Overlap Cohort. BioMed Res. Int..

[121] Bhansing K.J., Lammens M., Knaapen H.K., Van Riel P.L., Van Engelen B.G., Vonk M.C. (2014). Scleroderma-polymyositis overlap syndrome versus idiopathic polymyositis and systemic sclerosis: A descriptive study on clinical features and myopathology. Arthritis Res. Ther..

[122] Landon-Cardinal O., Baril-Dionne A., Hoa S., Meyer A., LeClair V., Bourré-Tessier J., Mansour A.-M., Zarka F., Makhzoum J.-P., Nehme J. (2020). Recognising the spectrum of scleromyositis: HEp-2 ANA patterns allow identification of a novel clinical subset with anti-SMN autoantibodies. RMD Open.

[123] An H., Tizaoui K., Terrazzino S., Cargnin S., Lee K., Nam S., Kim J., Yang J., Lee J., Smith L. (2020). Sarcopenia in Autoimmune and Rheumatic Diseases: A Comprehensive Review. Int. J. Mol. Sci..

[124] Cruz-Jentoft A.J., Baeyens J.P., Bauer J.M., Boirie Y., Cederholm T., Landi F., Martin F.C., Michel J.-P., Rolland Y., Schneider S.M. (2010). Sarcopenia: European consensus on definition and diagnosis: Report of the European Working Group on Sarcopenia in Older People. Age Ageing.

[125] Dalle S., Rossmeislova L., Koppo K. (2017). The Role of Inflammation in Age-Related Sarcopenia. Front. Physiol..

[126] Beyer I., Mets T., Bautmans I. (2012). Chronic low-grade inflammation and age-related sarcopenia. Curr. Opin. Clin. Nutr. Metab. Care.

[127] Marighela T.F., Genaro P.D.S., Pinheiro M.M., Szejnfeld V.L., Kayser C. (2013). Risk factors for body composition abnormalities in systemic sclerosis. Clin. Rheumatol..

[128] Cruz-Jentoft A.J., Bahat G., Bauer J., Boirie Y., Bruyère O., Cederholm T., Cooper C., Landi F., Rolland Y., Sayer A.A. (2019). Writing Group for the European Working Group on Sarcopenia in Older People 2 (EWGSOP2), and the Extended Group for EWGSOP2. Sarcopenia: Revised European consensus on definition and diagnosis. Age Ageing.

[129] Caimmi C., Caramaschi P., Venturini A., Bertoldo E., Vantaggiato E., Viapiana O., Ferrari M., Lippi G., Frulloni L., Rossini M. (2017). Malnutrition and sarcopenia in a large cohort of patients with systemic sclerosis. Clin. Rheumatol..

[130] Siegert E., March C., Otten L., Makowka A., Preis E., Buttgereit F., Riemekasten G., Müller-Werdan U., Norman K. (2018). Prevalence of sarcopenia in systemic sclerosis: Assessing body composition and functional disability in patients with systemic sclerosis. Nutrition.

[131] Corallo C., Fioravanti A., Tenti S., Pecetti G., Nuti R., Giordano N. (2019). Sarcopenia in systemic sclerosis: The impact of nutritional, clinical, and laboratory features. Rheumatol. Int..

[132] Paolino S., Goegan F., Cimmino M.A., Casabella A., Pizzorni C., Patanè M., Schenone C., Tomatis V., Sulli A., Gotelli E. (2020). Advanced microvascular damage associated with occurence of sarcopenia in systemic sclerosis patients: Results from a retrospective cohort study. Clin. Exp. Rheumatol..

[133] Hax V., Santo R.C.D.E., dos Santos L.P., Farinon M., de Oliveira M.S., Três G.L., Gasparin A.A., de Andrade N.P.B., Bredemeier M., Xavier R.M. (2021). Practical screening tools for sarcopenia in patients with systemic sclerosis. PLoS ONE.

[134] Doerfler B., Allen T.S., Southwood C., Brenner D., Hirano I., Sheean P. (2015). Medical Nutrition Therapy for Patients With Advanced Systemic Sclerosis (MNT PASS): A Pilot Intervention Study. J. Parenter. Enter. Nutr..

[135] Cen X., Feng S., Wei S., Yan L., Sun L. (2020). Systemic sclerosis and risk of cardiovascular disease: A PRISMA-compliant systemic review and meta-analysis of cohort studies. Medicine.

[136] Elhai M., Meune C., Boubaya M., Avouac J., Hachulla E., Balbir-Gurman A., Riemekasten G., Airã P., Joven B., Vettori S. (2017). Mapping and predicting mortality from systemic sclerosis. Ann. Rheum. Dis..

[137] Jaeger V., Wirz E.G., Allanore Y., Rossbach P., Riemekasten G., Hachulla E., Distler O., Airò P., Carreira P.E., Gurman A.B. (2016). Incidences and Risk Factors of Organ Manifestations in the Early Course of Systemic Sclerosis: A Longitudinal EUSTAR Study. PLoS ONE.

[138] Allanore Y., Meune C., Vonk M., Airo P., Hachulla E., Caramaschi P., Riemekasten G., Cozzi F., Beretta L., Derk C.T. (2009). Prevalence and factors associated with left ventricular dysfunction in the EULAR Scleroderma Trial and Research group (EUSTAR) database of patients with systemic sclerosis. Ann. Rheum. Dis..

[139] Parks J.L., Taylor M.H., Parks L.P., Silver R.M. (2014). Systemic Sclerosis and the Heart. Rheum. Dis. Clin. N. Am..

[140] Ross L., Prior D., Proudman S., Vacca A., Baron M., Nikpour M. (2019). Defining primary systemic sclerosis heart involvement: A scoping literature review. Semin. Arthritis Rheum..

[141] Krumm P., Mueller K.A.L., Klingel K., Krämer U., Horger M.S., Zitzelsberger T., Kandolf R., Gawaz M., Nikolaou K., Klumpp B.D. (2016). Cardiovascular magnetic resonance patterns of biopsy proven cardiac involvement in systemic sclerosis. J. Cardiovasc. Magn. Reson..

[142] Sandmeier B., Jaeger V., Nagy G., Carreira P.E., Tzankov A., Widuchowska M., Antic M., Distler O., Reichert H., Distler J.H.W. (2015). Autopsy versus clinical findings in patients with systemic sclerosis in a case series from patients of the EUSTAR database. Clin. Exp. Rheumatol..

[143] Bournia V.-K., Tountas C., Protogerou A.D., Panopoulos S., Mavrogeni S., Sfikakis P.P. (2018). Update on assessment and management of primary cardiac involvement in systemic sclerosis. J. Scleroderma Relat. Disord..

[144] Bruni C., Ross L. (2021). Cardiac involvement in systemic sclerosis: Getting to the heart of the matter. Best Pract. Res. Clin. Rheumatol..

[145] Allanore Y., Meune C. (2010). Primary myocardial involvement in systemic sclerosis: Evidence for a microvascular origin. Clin. Exp. Rheumatol..

[146] Giucă A., Gegenava T., Mihai C.M., Jurcuţ C., Săftoiu A., Gȋrniţă D.M., Popescu B.A., Marsan N.A., Jurcuț R. (2022). Sclerodermic Cardiomyopathy—A State-of-the-Art Review. Diagnostics.

[147] D’Angelo W., Fries J., Masi A., Shulman L. (1969). Pathologic observations in systemic sclerosis (scleroderma). A study of fifty-eight autopsy cases and fifty-eight matched controls. Am. J. Med..

[148] Bulkley B.H., Ridolfi R.L., Salyer W.R., Hutchins G.M. (1976). Myocardial lesions of progressive systemic sclerosis. A cause of cardiac dysfunction. Circulation.

[149] Murata I., Takenaka K., Shinohara S., Suzuki T., Sasaki T., Yamamoto K. (1998). Diversity of myocardial involvement in systemic sclerosis: An 8-year study of 95 Japanese patients. Am. Heart J..

[150] Fernandes F., Ramires F.J.A., Arteaga E., Ianni B.M., Bonfá E.S.D.O., Mady C. (2003). Cardiac remodeling in patients with systemic sclerosis with no signs or symptoms of heart failure: An endomyocardial biopsy study. J. Card. Fail..

[151] Mueller K.A.L., Mueller I.I., Eppler D., Zuern C., Seizer P., Kramer U., Koetter I., Roecken M., Kandolf R., Gawaz M. (2015). Clinical and Histopathological Features of Patients with Systemic Sclerosis Undergoing Endomyocardial Biopsy. PLoS ONE.

[152] De Luca G., Campochiaro C., De Santis M., Sartorelli S., Peretto G., Sala S., Canestrari G., De Lorenzis E., Basso C., Rizzo S. (2020). Systemic sclerosis myocarditis has unique clinical, histological and prognostic features: A comparative histological analysis. Rheumatology.

[153] Sano M., Satoh H., Suwa K., Nobuhara M., Saitoh T., Saotome M., Urushida T., Katoh H., Shimoyama K., Suzuki D. (2014). Characteristics and clinical relevance of late gadolinium enhancement in cardiac magnetic resonance in patients with systemic sclerosis. Hear. Vessel..

[154] Rodríguez-Reyna T.S., Morelos-Guzman M., Hernández-Reyes P., Montero-Duarte K., Martínez-Reyes C., Reyes-Utrera C., Madrid J.V.-L., Morales-Blanhir J., Núñez-Álvarez C., Cabiedes-Contreras J. (2014). Assessment of myocardial fibrosis and microvascular damage in systemic sclerosis by magnetic resonance imaging and coronary angiotomography. Rheumatology.

[155] Mavrogeni S.I., Bratis K., Karabela G., Spiliotis G., Van Wijk K., Hautemann D., Reiber J.H.C., Koutsogeorgopoulou L., Markousis-Mavrogenis G., Kolovou G. (2015). Cardiovascular Magnetic Resonance Imaging clarifies cardiac pathophysiology in early, asymptomatic diffuse systemic sclerosis. Inflamm. Allergy-Drug Targets.

[156] Barison A., Gargani L., De Marchi D., Aquaro G.D., Guiducci S., Picano E., Cerinic M.M., Pingitore A. (2014). Early myocardial and skeletal muscle interstitial remodelling in systemic sclerosis: Insights from extracellular volume quantification using cardiovascular magnetic resonance. Eur. Hear. J. Cardiovasc. Imaging.

[157] Mavrogeni S., Karabela G., Koutsogeorgopoulou L., Stavropoulos E., Katsifis G., Plastiras S.C., Kitas G.D., Panopoulos S., Pentazos G., Tzatzaki E. (2016). Pseudo-infarction pattern in diffuse systemic sclerosis. Evaluation using cardiovascular magnetic resonance. Int. J. Cardiol..

[158] Kobayashi Y., Kobayashi H., Giles J.T., Yokoe I., Hirano M., Nakajima Y., Takei M. (2016). Detection of Left Ventricular Regional Dysfunction and Myocardial Abnormalities Using Complementary Cardiac Magnetic Resonance Imaging in Patients with Systemic Sclerosis without Cardiac Symptoms: A Pilot Study. Intern. Med..

[159] Meduri A., Di Molfetta D.V., Natale L., Manfredi R. (2017). Cardiac magnetic resonance in systemic sclerosis patients with cardiac symptoms. Eur. Rev. Med Pharmacol. Sci..

[160] Mavrogeni S., Koutsogeorgopoulou L., Karabela G., Stavropoulos E., Katsifis G., Raftakis J., Plastiras S., Noutsias M., Markousis-Mavrogenis G., Kolovou G. (2017). Silent myocarditis in systemic sclerosis detected by cardiovascular magnetic resonance using Lake Louise criteria. BMC Cardiovasc. Disord..

[161] Hromádka M., Seidlerová J., Suchý D., Rajdl D., Lhotský J., Ludvík J., Rokyta R., Baxa J. (2017). Myocardial fibrosis detected by magnetic resonance in systemic sclerosis patients—Relationship with biochemical and echocardiography parameters. Int. J. Cardiol..

[162] Giacomelli R., Di Cesare E., Cipriani P., Ruscitti P., Di Sibio A., Liakouli V., Gennarelli A., Carubbi F., Splendiani A., Berardicurti O. (2017). Pharmacological stress, rest perfusion and delayed enhancement cardiac magnetic resonance identifies very early cardiac involvement in systemic sclerosis patients of recent onset. Int. J. Rheum. Dis..

[163] Muresan L., Oancea I., Mada R.O., Petcu A., Pamfil C., Muresan C., Rinzis M., Pop D., Zdrenghea D., Rednic S. (2018). Relationship Between Ventricular Arrhythmias, Conduction Disorders, and Myocardial Fibrosis in Patients With Systemic Sclerosis. JCR J. Clin. Rheumatol..

[164] Lee D.C., Hinchcliff M.E., Sarnari R., Stark M.M., Lee J., Koloms K., Hoffmann A., Carns M., Thakrar A., Aren K. (2018). Diffuse cardiac fibrosis quantification in early systemic sclerosis by magnetic resonance imaging and correlation with skin fibrosis. J. Scleroderma Relat. Disord..

[165] Gyllenhammar T., Kanski M., Engblom H., Wuttge D.M., Carlsson M., Hesselstrand R., Arheden H. (2018). Decreased global myocardial perfusion at adenosine stress as a potential new biomarker for microvascular disease in systemic sclerosis: A magnetic resonance study. BMC Cardiovasc. Disord..

[166] Bissell L.A., Dumitru R.B., Erhayiem B., Abignano G., Fent G., Kidambi A., Donica H., Burska A., Del Galdo F., Biglands G. (2019). Incidental significant arrhythmia in scleroderma associates with cardiac magnetic resonance measure of fibrosis and hs-TnI and NT-proBNP. Rheumatology.

[167] Tipparot T., Foocharoen C., Mahakkanukrauh A., Suwannaroj S., Nanagara R., Pussadhamma B., Chaosuwannakit N. (2019). Clinical and laboratory predictions of myocardial inflammation as detected by cardiac magnetic resonance imaging in patients with systemic sclerosis: A pilot study. Int. J. Rheum. Dis..

[168] Sugiyama K., Kobayashi H., Kobayashi Y., Yokoe I., Takei M., Kitamura N. (2019). Association of cardiac magnetic resonance-detected myocardial abnormalities with disease characteristics and brain natriuretic peptide levels in systemic sclerosis without cardiac symptoms. Int. J. Rheum. Dis..

[169] Rodríguez-Reyna T.S., Rosales-Uvera S.G., Kimura-Hayama E., Hernández-Reyes P., Mercado-Velázquez P., Benavides-Suárez S.A., Esquinca-González A., Núñez-Álvarez C.A. (2019). Myocardial fibrosis detected by magnetic resonance imaging, elevated U-CRP and higher mRSS are predictors of cardiovascular complications in systemic sclerosis (SSc) patients. Semin. Arthritis Rheum..

[170] Markousis-Mavrogenis G., Bournia V.-K., Panopoulos S., Koutsogeorgopoulou L., Kanoupakis G., Apostolou D., Katsifis G., Polychroniadis M., Dimitroulas T., Kolovou G. (2019). Cardiovascular Magnetic Resonance Identifies High-Risk Systemic Sclerosis Patients with Normal Echocardiograms and Provides Incremental Prognostic Value. Diagnostics.

[171] Gigante A., Galea N., Borrazzo C., Tubani L., Liberatori M., Ciolina F., Fiorelli A., Romaniello A., Barbano B., Romaggioli L. (2019). Role of autonomic dysfunction in the regulation of myocardial blood flow in systemic sclerosis evaluated by cardiac magnetic resonance. Int. J. Rheum. Dis..

[172] Bratis K., Lindholm A., Hesselstrand R., Arheden H., Karabela G., Stavropoulos E., Katsifis G., Kolovou G., Kitas G.D., Sfikakis P.P. (2019). CMR feature tracking in cardiac asymptomatic systemic sclerosis: Clinical implications. PLoS ONE.

[173] Terrier B., Dechartres A., Gouya H., Ben Arfi M., Bérézne A., Régent A., Dunogué B., London J., Cohen P., Guillevin L. (2020). Cardiac Intravoxel Incoherent Motion Diffusion-Weighted Magnetic Resonance Imaging With T1 Mapping to Assess Myocardial Perfusion and Fibrosis in Systemic Sclerosis: Association With Cardiac Events From a Prospective Cohort Study. Arthritis Rheumatol..

[174] Poindron V., Chatelus E., Canuet M., Gottenberg J.-E., Arnaud L., Gangi A., Gavand P.-E., Guffroy A., Korganow A.-S., Germain P. (2019). T1 mapping cardiac magnetic resonance imaging frequently detects subclinical diffuse myocardial fibrosis in systemic sclerosis patients. Semin. Arthritis Rheum..

[175] Mavrogeni S., Gargani L., Pepe A., Monti L., Markousis-Mavrogenis G., De Santis M., De Marchi D., Koutsogeorgopoulou L., Karabela G., Stavropoulos E. (2019). Cardiac magnetic resonance predicts ventricular arrhythmias in scleroderma: The Scleroderma Arrhythmia Clinical Utility Study (SAnCtUS). Rheumatology.

[176] Markousis-Mavrogenis G., Koutsogeorgopoulou L., Katsifis G., Dimitroulas T., Kolovou G., Kitas G.D., Sfikakis P.P., Mavrogeni S.I. (2020). The Double-Edged Sword of T1-Mapping in Systemic Sclerosis—A Comparison with Infectious Myocarditis Using Cardiovascular Magnetic Resonance. Diagnostics.

[177] Galea N., Rosato E., Gigante A., Borrazzo C., Fiorelli A., Barchetti G., Trombetta A.C., DiGiulio M.A., Francone M., Catalano C. (2020). Early myocardial damage and microvascular dysfunction in asymptomatic patients with systemic sclerosis: A cardiovascular magnetic resonance study with cold pressor test. PLoS ONE.

[178] Bordonaro V., Bivort D., Dresselaers T., De Langhe E., Bogaert J., Symons R. (2020). Myocardial T1 mapping and extracellular volume quantification as novel biomarkers in risk stratification of patients with systemic sclerosis. Clin. Radiol..

[179] Hromadka M., Baxa J., Seidlerova J., Miklik R., Rajdl D., Sudova V., Suchy D., Rokyta R. (2021). Myocardial Involvement Detected Using Cardiac Magnetic Resonance Imaging in Patients with Systemic Sclerosis: A Prospective Observational Study. J. Clin. Med..

[180] Dumitru R.B., Bissell L.-A., Erhayiem B., Fent G., Kidambi A., Swoboda P., Abignano G., Donica H., Burska A., Greenwood J.P. (2020). Predictors of subclinical systemic sclerosis primary heart involvement characterised by microvasculopathy and myocardial fibrosis. Rheumatology.

[181] Dumitru R.B., Bissell L.-A., Erhayiem B., Kidambi A., Dumitru A.-M.H., Fent G., Abignano G., Donica H., Burska A., Greenwood J.P. (2021). Cardiovascular outcomes in systemic sclerosis with abnormal cardiovascular MRI and serum cardiac biomarkers. RMD Open.

[182] Vos J.L., Butcher S.C., Fortuni F., Galloo X., Rodwell L., Vonk M.C., Bax J.J., van Leuven S.I., de Vries-Bouwstra J.K., Snoeren M. (2022). The Prognostic Value of Right Atrial and Right Ventricular Functional Parameters in Systemic Sclerosis. Front. Cardiovasc. Med..

[183] Palumbo P., Ruscitti P., Cannizzaro E., Berardicurti O., Conforti A., Di Cesare A., Di Cola I., Giacomelli R., Splendiani A., Barile A. (2022). Unenhanced Cardiac Magnetic Resonance may improve detection and prognostication of an occult heart involvement in asymptomatic patients with systemic sclerosis. Sci. Rep..

[184] Knight D.S., Karia N., Cole A.R., Maclean R.H., Brown J.T., Masi A., Patel R.K., Razvi Y., Chacko L., Venneri L. (2022). Distinct cardiovascular phenotypes are associated with prognosis in systemic sclerosis: A cardiovascular magnetic resonance study. Eur. Heart J.—Cardiovasc. Imaging.

